# Mapping Human Corpus Callosum Connectivity With Diffusion Spectrum Imaging: A Deterministic Tractography Approach

**DOI:** 10.1002/brb3.71306

**Published:** 2026-03-12

**Authors:** Chao Zhang, Jian‐Wei Shi, Zhen‐Ming Wang, Peng‐Hu Wei, Chao Lu, Yi‐He Wang, Hua‐Qiang Zhang, Xiao‐Tong Fan, Yong‐Zhi Shan, Jie Lu, Si‐Qi Ou, Guo‐Guang Zhao

**Affiliations:** ^1^ Department of Neurosurgery, Xuanwu Hospital Capital Medical University Beijing China; ^2^ China International Neuroscience Institute (China‐INI) Beijing China; ^3^ Clinical Research Center For Epilepsy Capital Medical University Beijing China; ^4^ Department of Radiology and Nuclear Medicine, Xuanwu Hospital Capital Medical University Beijing China; ^5^ Beijing Key Laboratory of Magnetic Resonance Imaging and Brain Informatics Beijing China; ^6^ Beijing Municipal Geriatric Medical Research Center Beijing China

**Keywords:** corpus callosum, diffusion spectrum imaging, deterministic tractography, transcallosal tracts

## Abstract

**Objective:**

To establish a high‐resolution atlas of the corpus callosum (CC) using diffusion spectrum imaging (DSI), aiming to detail the subregional connectivity and improve the understanding of interhemispheric communication for clinical applications.

**Methods:**

This research employed DSI in conjunction with quantitative anisotropy (QA)‐based deterministic fiber tracking on 44 healthy individuals to map the connectivity patterns of the CC, correlating these with cortical subregions defined in the automated anatomical labeling (AAL) and human brainnetome atlas (BNA).

**Results:**

The study identified 41 regions corresponding to the AAL atlas and 101 regions related to the BNA atlas at the midsagittal plane of the CC. Specifically, it included 34 frontal subregions associated with higher brain functions located predominantly in the anterior part of the CC. The midbody of the CC harbored subregions related to primary motor and sensory functions, while the splenium was characterized by subregions containing temporal projections. This comprehensive mapping revealed a complex and nuanced connectivity pattern within the CC, highlighting significant heterogeneity across regions that reflects its diverse structural and functional roles in brain functionality.

**Conclusion:**

The developed atlas represents the first extensive mapping of the CC integrating both anatomical and functional connectivity paradigms, using QA‐based DSI deterministic tractography. This atlas, which will be freely available, provides a valuable resource for neuroscientific research and clinical practice, offering detailed insights into the structural and functional organization of the CC.

## Graphical abstract

Our study developed a callosal template incorporating macro‐anatomic and long‐range connectional information, providing detailed transcallosal tracts and the corresponding topography of callosal subregions at high resolution using DSI techniques. In addition, the QA‐based structural connectome observed in this study reflects the relationship between patterns of interhemispheric connectivity and cortical development.

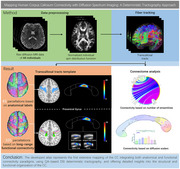

## 1 Introduction

The human corpus callosum (CC) is the largest white matter fiber bundle in the brain, connecting most of the cortical regions in both hemispheres and comprising more than 200 million commissural fibers (Tomasch 1954). It plays an important role in communication among and integration of interhemispheric transcallosal network (ITN), including those related to both low‐order (e.g., visual, motor, and sensory) and high‐order functions (e.g., association and cognition) (Schmahmann et al. 2006). However, previous studies have emphasized gray matter over white matter, which has curtailed advancements in clinical practices for preoperative assessments and prognosis of white matter‐related diseases (Bae et al. 2020). As the functional properties and specialization of a given cortical subregion are jointly determined by local cytoarchitecture and long‐range connectivity (Eickhoff and Grefkes 2011), a subdivision of the CC connecting a certain cortical subregion may exhibit its corresponding characteristics in terms of fiber microstructure, which may contribute to its connectivity with the cortex. Precise mapping of these relationships may allow us to determine the corresponding cortical functional properties of a given callosal subregion, offering valuable insights for clinical applications.

Research has shown the CC to be a compact, heterogeneous bundle without clear macroscopic landmarks or definitive boundaries (Aboitiz and Montiel 2003; Aboitiz et al. 1992). Its microstructural and functional organization exhibits significant heterogeneity, with callosal fibers differing in diameter, myelination, and conduction velocity based on the specific cortical subregions they connect. This heterogeneity likely reflects adaptations to meet the distinct functional demands of different cortical areas, such as sensory processing, motor coordination, and higher‐order cognitive functions. Despite its importance, comprehensive quantification and characterization of this heterogeneity remain lacking (Taylor and Forsyth 2016). Conventional neural tracing and microsurgical techniques are limited in humans due to anatomical complexities (Boussaoud et al. 2005; Fernández‐Miranda et al. 2008). Thus far, Witelson (1989) proposed the most widely applied scheme, in which the CC is subjectively parcellated into seven regions with rigid boundaries. To overcome the shortcomings of parcellation based on histology or geometry alone, parcellation schemes using diffusion‐weighted magnetic resonance imaging (dMRI) have been proposed and implemented into primary connectome‐based atlases (Abe et al. 2004; Chao et al. 2009; Hofer and Frahm 2006; Huang et al. 2005; Pannek et al. 2010; Park et al. 2008). However, the accuracy of diffusion tensor imaging (DTI) in tracking complex fiber crossings remains inadequate due to technical constraints (Jones 2008; Wei et al. 2017). Current DTI studies suggest that CC divisions based on functional connectivity do not align well with specific cortical anatomical structures (Friedrich, Forkel et al. 2020; Wang et al. 2021), and existing atlases like the Brodmann and automated anatomical labeling (AAL) fail to accurately reflect the functional aspects of cortical‐callosal relationships (Archer et al. 2019; Chao et al. 2009). To be appropriate for surgical use, a callosal atlas must accurately define the relationship between the location of callosal fibers and the functional properties of the corresponding cortical subregions.

Diffusion spectrum imaging (DSI)‐derived connectomics analyses are pivotal for crafting a nuanced callosal atlas tailored for surgical interventions (Jeurissen et al. 2019). Quantitative anisotropy (QA)‐based deterministic tractography facilitates the precise delineation of fiber constitution at the voxel level, enabling the accurate reconstruction of intricate white‐matter structures (Yeh et al. 2013). Superior to traditional diffusion techniques such as DTI and high angular resolution diffusion imaging (HARDI), QA‐enhanced DSI offers greater fidelity in mapping structural connectomes, making it more suitable for clinical and surgical applications (Tuch et al. 2002; Yeh and Verstynen 2016; Yeh et al. 2013). Furthermore, the integration of comprehensive cortical atlases, notably the human brainnetome atlas (BNA), enriches our understanding of the interplay between cortical structures and callosal connectivity, serving as a foundational tool in refining callosal mappings (Fan et al. 2016; Rolls et al. 2020). A finer functional and anatomical division of the CC can be applicable for surgeons when attempting precise thermal therapy for lesions within the CC (Beaumont et al. 2018) or prospective neuromodulation through the transcallosal tracts (Couturier and Durand 2018).

Therefore, this study had two objectives. First, by mapping the cortical subregions identified in the BNA and the initial macro‐anatomic framework (AAL3) to the CC, we aimed to create a fine‐grained callosal template to define the callosal subdivisions corresponding to these cortical regions (i.e., callosal topography). Additionally, we designed a quantitative analysis including indices of streamline number and diffusion scalars to delineate the structural connectome of the ITN and explore the characteristics of callosal fibers, such as microarchitecture and the resulting connectivity.

## 2 Methods

### 2.1 Study Design

**FIGURE 1:**
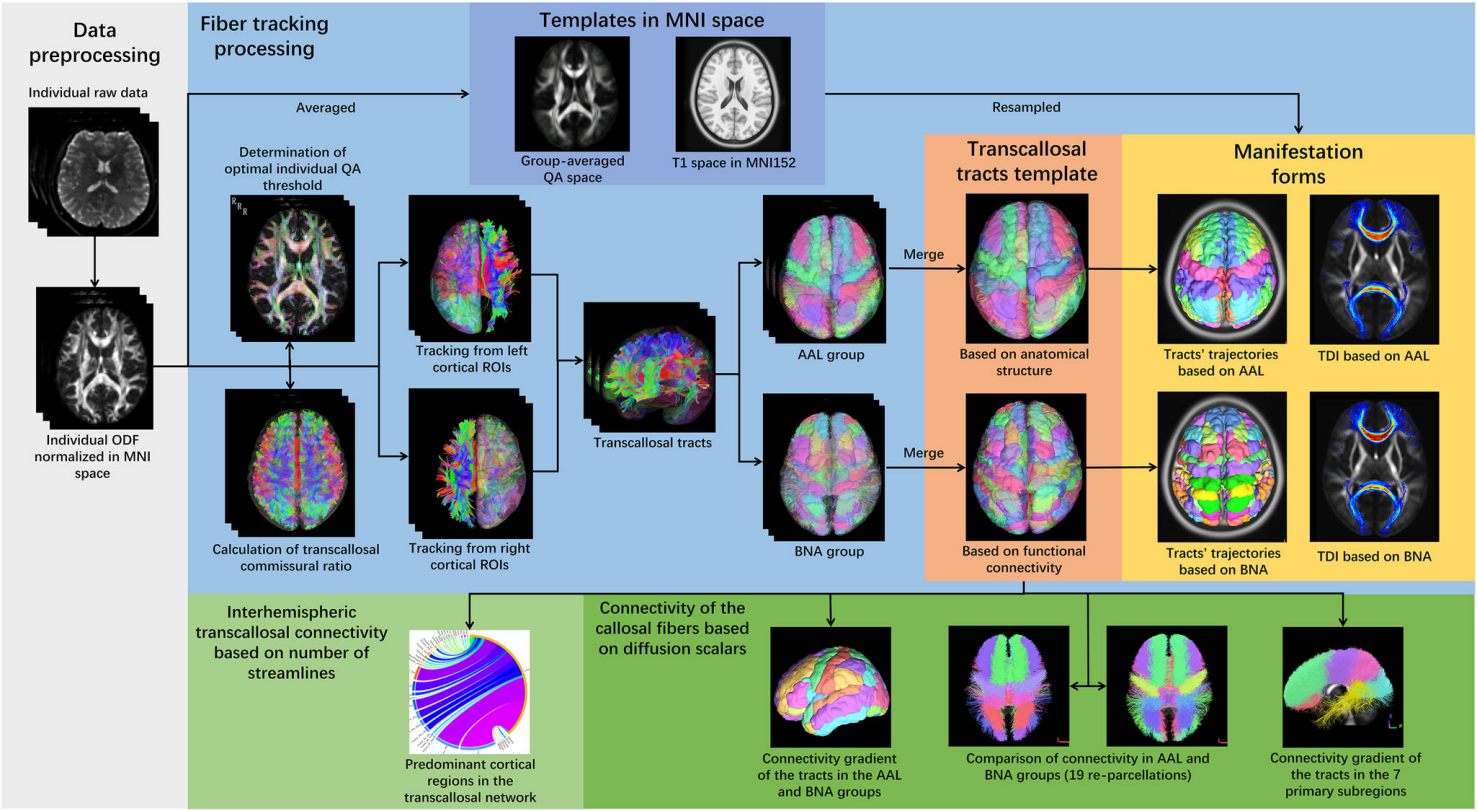
Flow chart of the data processing and analysis. Preprocessing of raw data (white panel) included warping the individual SDF to the MNI space to create normalized individual SDF maps and a group‐averaged QA space. Processing of fiber tracking (bluish panel) included determination of optimal QA threshold, calculation of transcallosal commissural ratio, fiber tracking, filter, and merging in individual QA spaces, to create transcallosal tracts templates based on anatomical labels and long‐range functional connectivity (red panel), respectively. The two templates were further mapped on the normalized T1 and QA spaces (blue violet panel) and manifested in forms of tracts’ trajectories and TDI (yellow panel). Further analysis included exploration of the interhemispheric transcallosal connectivity (prasinous panel) and connectivity of the callosal fibers (green panel). AAL, automated anatomical labeling atlas; BNA, Human Brainnetome Atlas; MNI, Montreal Neurological Institute; QA, quantitative anisotropy; SDF, spin distribution function; TDI, track‐density image.

A dataset with 44 groups of structural and diffusion images from healthy young volunteers was analyzed first. We reconstructed the spin distribution function (SDF) of all individuals and projected them to the Montreal Neurological Institute (MNI) space to create a group‐averaged SDF template. Then, the transcallosal fibers of the cerebral homologous regions parcellated using anatomical and functional atlases were tracked in each individual normalized QA space and projected to the group‐averaged template. Finally, transcallosal tract templates and refined callosal topographies were estimated based on cortical anatomical and functional connectivity, and a quantitative analysis was performed to evaluate the transcallosal connectome. The workflow is shown in Figure 1.

### 2.2 Ethics Approval and Volunteers' Consent Statement

This study was approved by the Ethical Committee of Xuanwu Hospital (No. LYS [2019] 097) and was conducted in accordance with the Declaration of Helsinki (as revised in 2013). All procedures followed were in accordance with the ethical standards of the responsible committee on human experimentation (institutional and national) and with the Helsinki Declaration of 1975, as revised in 2000. Informed consent was obtained from all volunteers to be included in the study.

### 2.3 Participants, Data Acquisition, and Preprocessing

From November 2021 to January 2023, 44 healthy young volunteers (age: 18–30 years, average age: 24.0 years; 16 men and 28 women) were recruited and underwent magnetic resonance imaging (MRI) examination. A brief description of the characteristics of the included participants is presented in Table S1.  Initially, 46 participants were recruited; however, two were excluded due to excessive head motion and image artifacts detected during quality control (see below), resulting in a final sample of 44. The MRI data were acquired at Xuanwu Hospital using a GE Signa Premier 3.0T scanner (General Electric Healthcare, Waukesha, WI, USA). Using a 64‐channel head coil, the half‐*q*‐space DSI data were acquired in 258 directions with maximum *b* values reaching 7000 s/mm^2^. All data were preprocessed using DSI‐Studio software. *Q*‐space diffeomorphic reconstruction (QSDR) is a model‐free reconstruction technique (Yeh and Tseng 2011). It is an extension of the generalized *q*‐sampling imaging (GQI) method that enables direct reconstruction of individual spin distribution functions (SDFs) within a standardized template space, such as the MNI space. Using this method, the SDFs of all participants were extracted and normalized to create individual SDF maps and a group‐averaged template. Consequently, we can perform group‐level comparisons and connectometry analyses. Quality control of data acquisition and preprocessing was independently performed by two experienced radiologists, following a standardized protocol that included visual inspection for motion and artifacts, verification of image orientation, and file integrity. Data that did not meet the quality standards were re‐acquired and pre‐processed. The details of recruitment are provided in Section S1.1. The parameters of MRI scanning, quality control of data acquisition, and preprocessing were provided in Section S1.2.

### 2.4 Fiber Tracking Process

Since directly applying a callosal mask extracted from T1 space may lead to bias in the individual QA space, we decided to label the CC as a region of interest (ROI) on the QA maps for all 44 individuals (see details in Section S1.3). Briefly, to define the ROI, two raters manually performed the segmentation of the mid‐sagittal CC using the same protocol. We used the intraclass correlation coefficient (ICC) for absolute agreement to quantify the consistency between two raters regarding the volume of the ROI and the voxel overlap rate on the QA map, thereby assessing the reliability of the ROI definition. The overlap rate was defined as the percentage of the overlapped region compared with the segmentation of the first rater. The area overlapped between the two raters was used as the ROI for fiber tracking.

Fiber tracking was conducted using DSI‐Studio software (Version: 2020_09. Details related to settings in Section S1.4). Here, as a robustness analysis, the transcallosal commissural ratio (Krupnik et al. 2021) was calculated to determine the optimal QA threshold for each participant. Since the number of streamlines was sensitive to the QA threshold set in fiber tracking, we first performed a robustness analysis to determine the optimal QA threshold for each individual. With the optimal QA threshold in the QA space, most of the fiber orientation signals were supposed to be in the white matter of the SDF map, while a few signals were in the subarachnoid space or ventricles as much as possible. In detail, we estimated the transcallosal commissural ratio, which was calculated as the percentage of transcallosal commissural tracts. The transcallosal fibers were tracked with the callosal masks as seeds, while the whole‐brain fibers were tracked by global seeding. The transcallosal commissural ratio was computed as the percentage of the transcallosal streamlines relative to the number of whole‐brain fibers in each individual QA space (*R_i_*), while the proportion in the single averaged QA template was represented by *R_a_*. Further, *R_i_* of 44 individuals and *R_a_* of a template were tested using one‐sample *t*‐test. Since most transcallosal commissures are relatively symmetrical (Clarke and Zaidel 1994; Jarbo et al. 2012), fibers that connect nonhomologous regions were filtered while interpreting the results. Here, we performed fiber tracking with region pairs of the left cortex (seed)‐CC (ROI) and right cortex (seed)‐CC (ROI), following which the fibers were merged to determine their trajectory at the CC as the regions to be plotted. The AAL3 is a classic anatomical parcellation of the cortex widely used in neuroimaging research (Rolls et al. 2020). The BNA is a novel scheme that “links brain connectivity to function,” and segments the cortex and subcortical gray matter based on long‐range functional connectivity, and names cortical subregions based on a combination of Brodmann's cytoarchitectonic divisions (Fan et al. 2016). Therefore, taking into account both the traditional anatomical marker‐based segmentation and cytoarchitectonic‐ and functional connectivity‐based segmentation, we selected these two atlases to delineate the cerebral cortex. We used the AAL3 (41 paired regions) as the template to define the projections at the macroscopic (i.e., gyrus) level, then used the BNA (105 paired regions) to localize the callosal subregions based on detailed functional parcellations. Subsequently, the distinct fibers of 44 participants were merged from the QSDR space of each individual to the single group‐averaged template for the AAL3 and BNA regions, respectively.

### 2.5 Visualization of the Transcallosal Tracts

To visualize fiber distribution, the pathways of the transcallosal tracts were transformed into “regions”. In addition, to display the most dense parts of these tracts at the group level, they were also transformed to the track‐density image (TDI) format, which provides a much higher spatial resolution for white matter images than common diffusion maps (e.g., fractional anisotropy [FA] maps) (Calamante et al. 2010). The resolution ratio was set to 8 (sub‐voxel size: 0.25 mm × 0.25 mm × 0.25 mm) (Dai et al. 2018). To enhance visualization of density variations across different callosal subregions, we scaled the colormap such that the maximum color intensity (crimson) corresponded to 30%–50% of the absolute maximum track‐density value in each region, rather than the absolute maximum, thereby improving the visibility of areas with moderate to low fiber density. The aforementioned data were projected to normalized spaces and presented using ITK‐SNAP 3.8.0 software, with details provided in Section S1.5. To facilitate the presentation of the segmentation pattern and enhance understanding, we presented the results of segmentation in the mid‐sagittal area of CC.

### 2.6 Connectome Analysis of ITN

To delineate the structural connectome of the ITN, we performed a quantitative analysis of transcallosal tracts from two perspectives.

First, in structural connectomics, the interhemispheric transcallosal connectivity (ITC) of homologous cortical regions is mostly reflected by the number of streamlines, which represents the richness of connections (Sporns 2014). In this analysis, the global connectivity matrices consisting of transcallosal fibers connecting all homologous cortical subregions in both hemispheres were presented with circular graphs, corresponding to the two adopted atlases, respectively. Afterwards, predominant cortical subregions with relatively larger proportions of streamlines were highlighted, respectively.

In addition, since QSDR quantifies the density of restricted and less‐restricted diffusion for each fiber (Yeh and Tseng 2011), restricted diffusion represents the constrained movement of water molecules caused by barriers such as axonal membranes, while less‐restricted diffusion reflects freer movement in extracellular spaces. These properties form the basis for calculating quantitative diffusion scalars, such as the normalized QA (NQA), which reflect fiber microarchitecture and callosal connectivity (Wang et al. 2020). The connectivity of various callosal fibers is reflected as a gradient distribution. However, since the number of cortical subdivisions differs between the two atlases (41 and 105 subdivisions, respectively), comparisons of diffusion scalars for the callosal fibers were not intuitive. Therefore, we utilized a re‐parcellation scheme (Section S1.6). Two experienced anatomists integrated the subregions from the two atlases into 19 structures with obvious macro‐anatomic features, which were paired between the two atlases (e.g., precentral gyrus, superior frontal gyrus, etc., as shown in Table S4). Subsequently, average NQA values of the callosal fibers corresponding to these 19 structures were extracted and tested using the paired *t*‐test or Wilcoxon test. The ICC between the two groups was also calculated using a two‐way random model.

Finally, to examine the organization of transcallosal connectivity at a macroscale level, we grouped the cortical subregions into seven functionally meaningful divisions commonly referenced in the literature: orbitofrontal cortex (OFC) (Rolls 2000), prefrontal cortex (PFC) (Fuster 2009), central region (encompassing the precentral and postcentral gyri) (Tzourio‐Mazoyer et al. 2002), parietal lobe, occipital lobe, temporal lobe, and insular lobe. This grouping consolidates the finer parcellations from the AAL3 and BNA into broader categories, facilitating comparison with prior callosal studies while maintaining sufficient fiber counts for reliable diffusion scalar analysis. For each of these seven divisions, we first merged the fibers into seven corresponding bundles and observed the proportion of transcallosal streamlines for each. Then, to delineate the gradient of callosal connectivity based on the NQA values, we tested the inter‐regional difference and attempted to divide this gradient using analyses of variance (ANOVA). All statistical analyses were performed using SPSS 22.0 (IBM Corp., Armonk, NY, USA) at a threshold of *p* < 0.05.

### 2.7 Transcallosal Tract Template and Callosal Topography

To obtain the topography of the callosal subregions, callosal fibers with lengths of 4 mm on the left and right sides of the plane, including the callosal midsection, were selected for further analysis. Using MATLAB and statistical parametric mapping (SPM) toolbox, both whole transcallosal tracts and the reserved callosal fibers, including 41 subdivisions corresponding to the AAL3 and 105 subdivisions corresponding to the BNA, were projected to the MNI space and integrated into 4D nifti format to create two groups of templates, respectively. The details are provided in Section **S1.7**.

These templates included the transcallosal tracts connecting the homologous cortical regions of the two atlases, as well as the topographies of corresponding callosal parcellations. Thus, all subregions in the CC corresponded to all cerebral cortical subregions and their functional properties. For each individual, the cortical regions and relevant functional properties corresponding to the fibers of a given callosal subregion, or to the location of the callosal subregion, were both defined by registering this template onto the individual T1 sequence.

### 2.8 Data and Code Availability Statement

The statistical data, code, and details pertaining to related software and atlases used in this study are provided in Section **S1.8**.

## 3 Results

**FIGURE 2:**
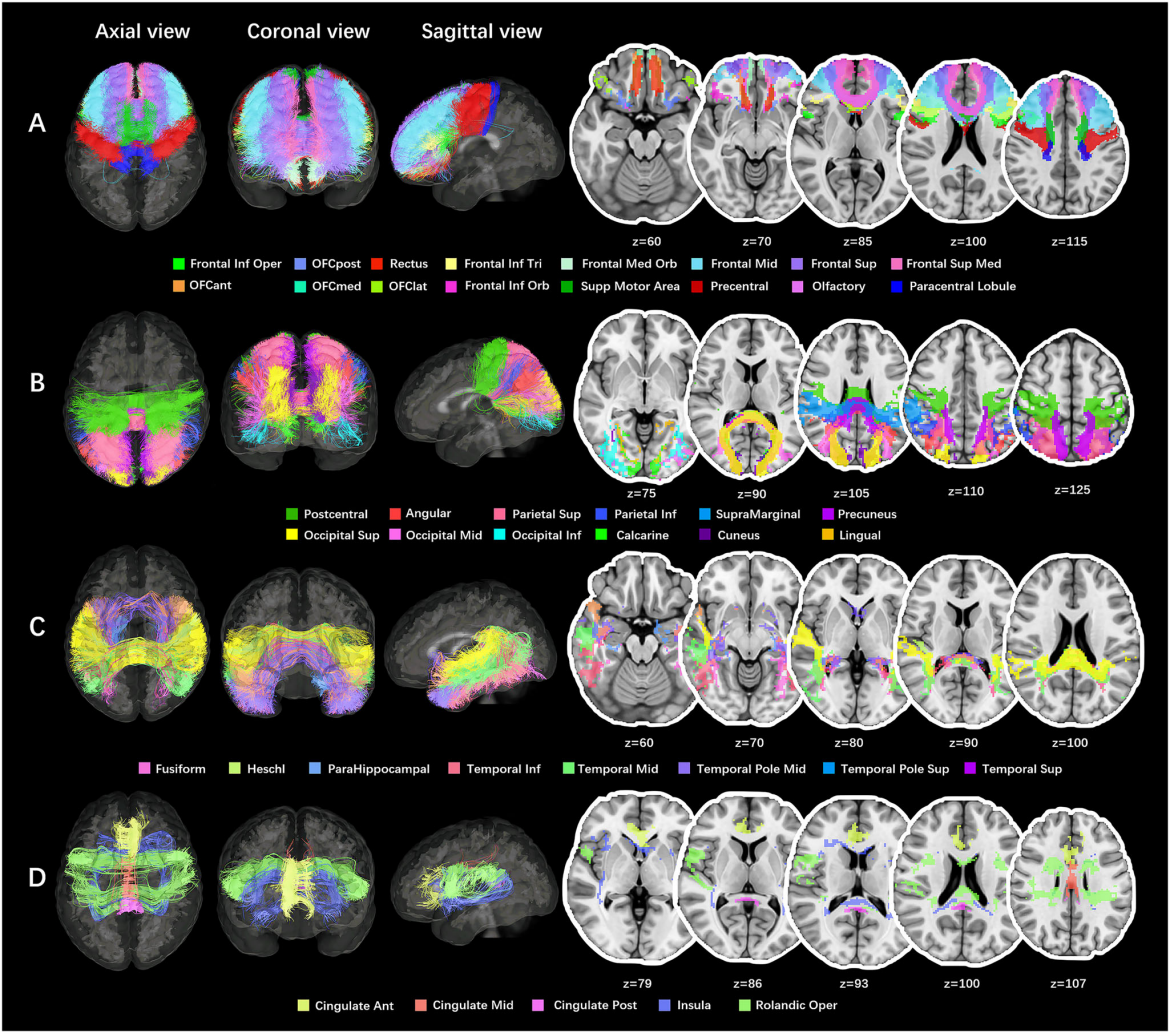
Pathways of the transcallosal tracts parcellated by the AAL3.The transcallosal tracts connecting homologous cortical regions in both hemispheres were parcellated into 41 parts based on the AAL3. These fibers were shown in the average QA space in three dimensions, while their trajectories (in “regions” format) were mapped on axial T1 sequences in MNI space. The transcallosal network included commissural fibers of 17 frontal areas (16 areas in panel A and 1 area in panel D [Rolandic Oper]), 6 parietal areas (B), 6 occipital areas (B), 8 temporal areas (C), 3 cingulate areas, and 1 insular area (D). AAL3, automated anatomical labeling atlas 3; Angular, angular gyrus; Calcarine, calcarine fissure and surrounding cortex; Cingulate Ant, anterior cingulate & paracingulate gyri; Cingulate Mid, middle cingulate & paracingulate gyri; Cingulate Post, posterior cingulate gyrus; Frontal Inf Oper, inferior frontal gyrus, opercular part; Frontal Inf Orb, inferior frontal gyrus, pars orbitalis; Frontal Inf Tri, inferior frontal gyrus, triangular part; Frontal Med Orb, superior frontal gyrus, medial orbital; Frontal Mid, middle frontal gyrus; Frontal Sup, superior frontal gyrus, dorsolateral; Frontal Sup Med, superior frontal gyrus, medial; Fusiform, fusiform gyrus; Heschl, Heschl's gyrus; Lingual, lingual gyrus; Occipital Inf, inferior occipital gyrus; Occipital Mid, middle occipital gyrus; Occipital Sup, superior occipital gyrus; OFCant, anterior orbital gyrus; OFClat, lateral orbital gyrus; OFCmed, medial orbital gyrus; OFCpost, posterior orbital gyrus; Olfactory, olfactory cortex; ParaHippocampal, parahippocampal gyrus; Parietal Inf, inferior parietal gyrus, excluding supramarginal and angular gyri; Parietal Sup, superior parietal gyrus; Postcentral, postcentral gyrus; Precentral, precentral gyrus; QA, quantitative anisotropy; Rectus, gyrus rectus; Rolandic Oper, Rolandic operculum; Supp Motor Area, supplementary motor area; SupraMarginal, supramarginal gyrus; Temporal Inf, inferior temporal gyrus; Temporal Mid, middle temporal gyrus; Temporal Pole Mid, Temporal pole: middle temporal gyrus; Temporal Pole Sup, temporal pole: superior temporal gyrus; Temporal Sup, superior temporal gyrus.

**FIGURE 3:**
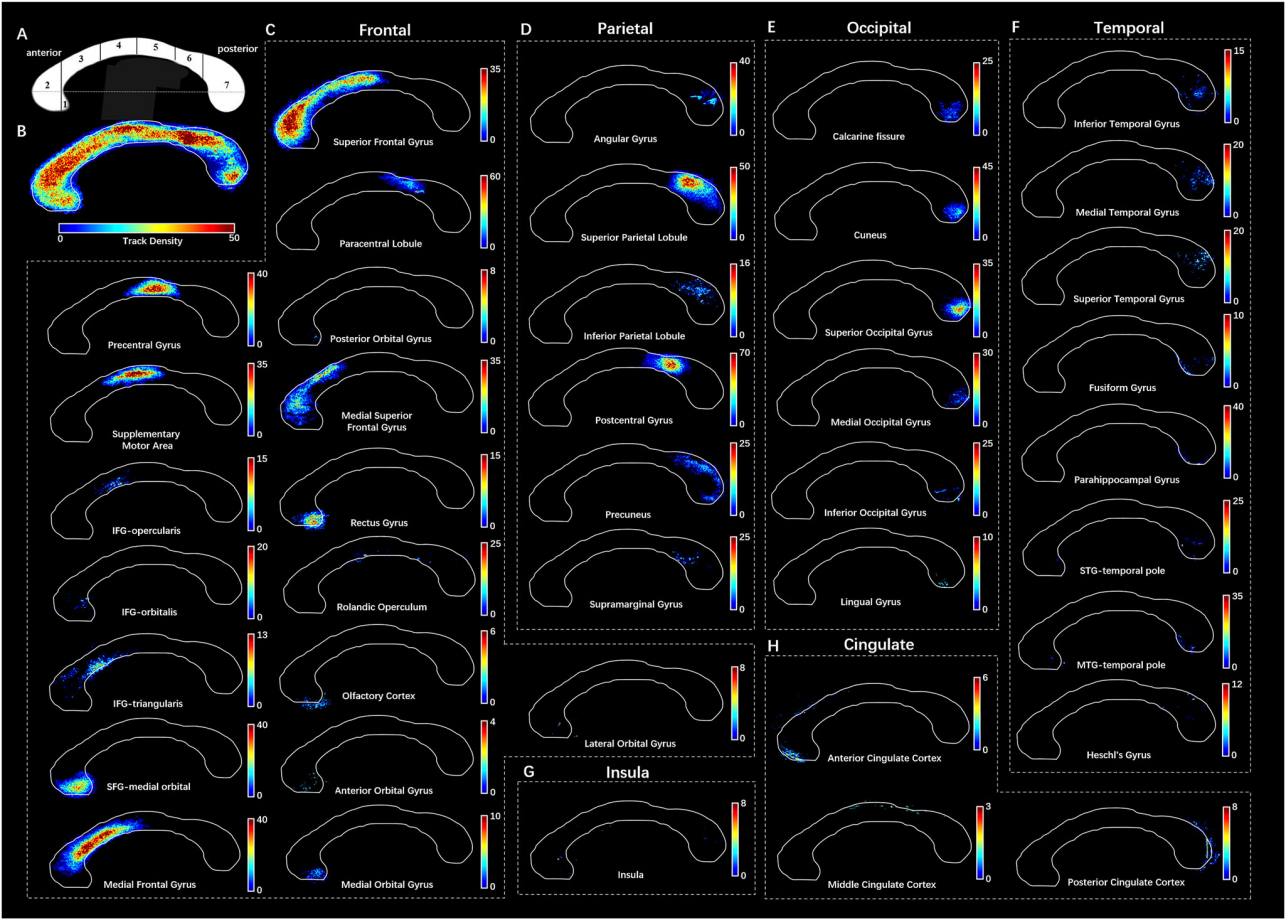
TDI of the transcallosal tracts parcellated by the AAL3.(A) Witelson (1989) parcellated the human CC into seven parts based on midsagittal geometrical characteristics, including the rostrum (1), genu (2), rostral body (3), anterior midbody (4), posterior midbody (5), isthmus (6) and splenium (7). The white dotted line connecting the callosal anteriormost and posteriormost points indicated the long axis of the CC. The profile of CC was outlined from the average QA template. (B) The TDI of all transcallosal tracts parcellated by AAL3 was plotted at the callosal midsection. The indigo‐crimson temperature map represented the density of fibers. The fibers were relatively denser in the dorsal genu, anterior rostral body, anterior midbody, posterior midbody, isthmus, and ventrocaudal splenium of the CC. (C–H) The callosal topography included 17 frontal areas (C), 6 parietal areas (D), 6 occipital areas (E), 8 temporal areas (F), 1 insular area (G), and 3 cingulate areas (H). AAL3, automated anatomical labeling atlas 3; CC, corpus callosum; IFG, inferior frontal gyrus; MTG, middle temporal gyrus; QA, quantitative anisotropy; STG, superior temporal gyrus; TDI, track‐density image.

**FIGURE 4:**
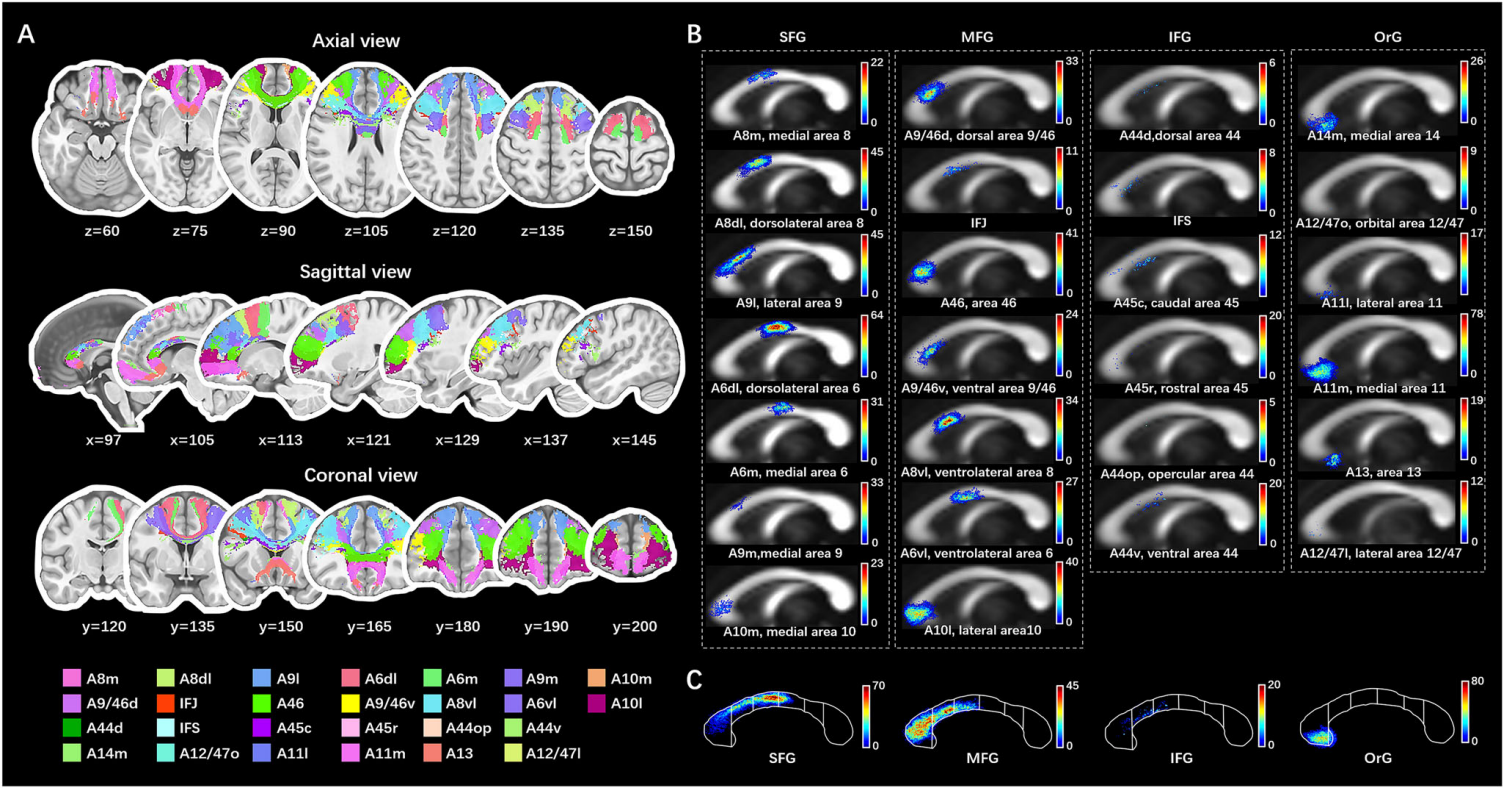
Trajectories and TDI of the prefrontal transcallosal tracts parcellated by the BNA.(A) Trajectories of the prefrontal transcallosal tracts parcellated based on the BNA were shown in 2‐dimensional T1 sequences in MNI space. The prefrontal lobe contained 7 subregions in the SFG, 7 subregions in the MFG, 6 subregions in the IFG, and 6 subregions in the OrG. (B and C) The fibers in TDI format were plotted at the callosal midsection. The subregions were named following the BNA's descriptions, which adopted the label schemes in the Brodmann atlas. (C) The subdivisions of the CC were segmented, referring to Witelson's scheme. In the PFC, the predominant cortical subregions with denser transcallosal fibers were mainly located at dorsolateral prefrontal cortex (DLPFC) (dorsolateral area 6, lateral area 8, dorsal area 9/46) and the frontal pole (FP) (lateral area 10 and medial area 11). BNA, human brainnetome atlas; CC, corpus callosum; IFG, inferior frontal gyrus; IFJ, inferior frontal junction; IFS, inferior frontal sulcus; MFG, middle frontal gyrus; OrG, orbital gyrus; SFG, superior frontal gyrus; TDI, track‐density image.

**FIGURE 5:**
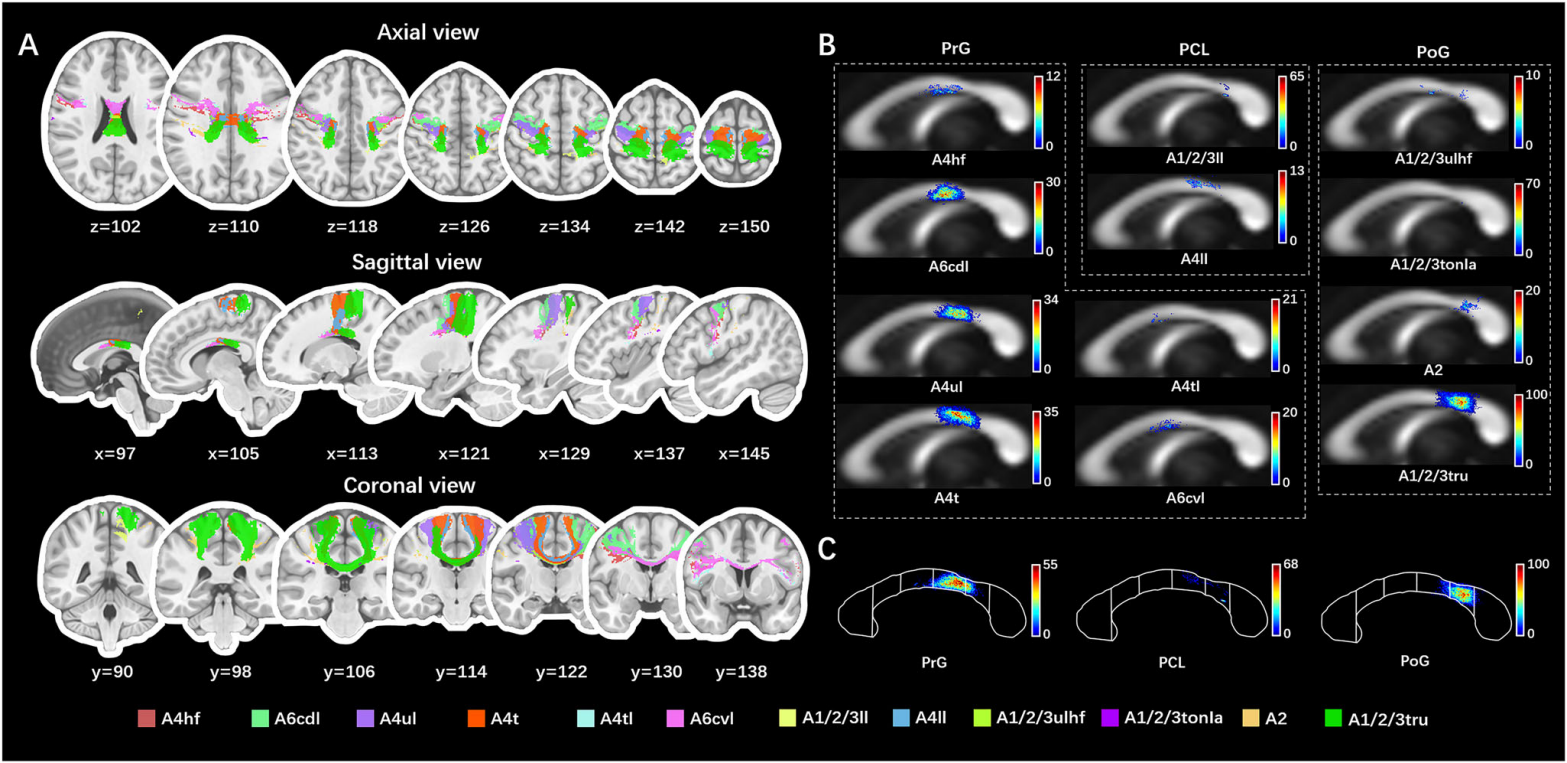
Trajectories and TDI of the transcallosal tracts corresponding to the central region (M1 and S1) parcellated by the BNA. (A) Trajectories of the transcallosal tracts, corresponding to the central region (M1 and S1) parcellated based on the BNA, were shown in 2‐dimensional T1 sequences in MNI space. The central region (M1 and S1) contained 6 subregions in the PrG, 2 subregions in the PCL, and 4 subregions in the PoG. (B and C) The fibers in TDI format were plotted at the callosal midsection. The subregions were named following the BNA's descriptions, which adopted the label schemes in the Brodmann atlas. (C) The subdivisions of the CC were segmented, referring to Witelson's scheme (Witelson 1989). For the central region, the predominant regions were caudal dorsolateral area 6, upper limb and trunk region of area 4, and trunk region of areas 1–3. A1/2/3ll, area 1/2/3 (lower limb region); A1/2/3tonIa, area 1/2/3 (tongue and larynx region); A1/2/3tru, area 1/2/3 (trunk region); A1/2/3ulhf, area 1/2/3 (upper limb, head and face region); A2, area 2; A4hf, area 4 (head and face region); A4ll, area 4 (lower limb region); A4t, area 4 (trunk region); A4tl, area 4 (tongue and larynx region); A4ul, area 4 (upper limb region); A6cdl, caudal dorsolateral area 6; A6cvl, caudal ventrolateral area 6; BNA, Human Brainnetome Atlas; CC, corpus callosum; M1, the primary motor cortex; PCL, paracentral lobule; PoG, postcentral gyrus; PrG, precentral gyrus; S1, the primary somatosensory cortex; TDI, track‐density image.

**FIGURE 6:**
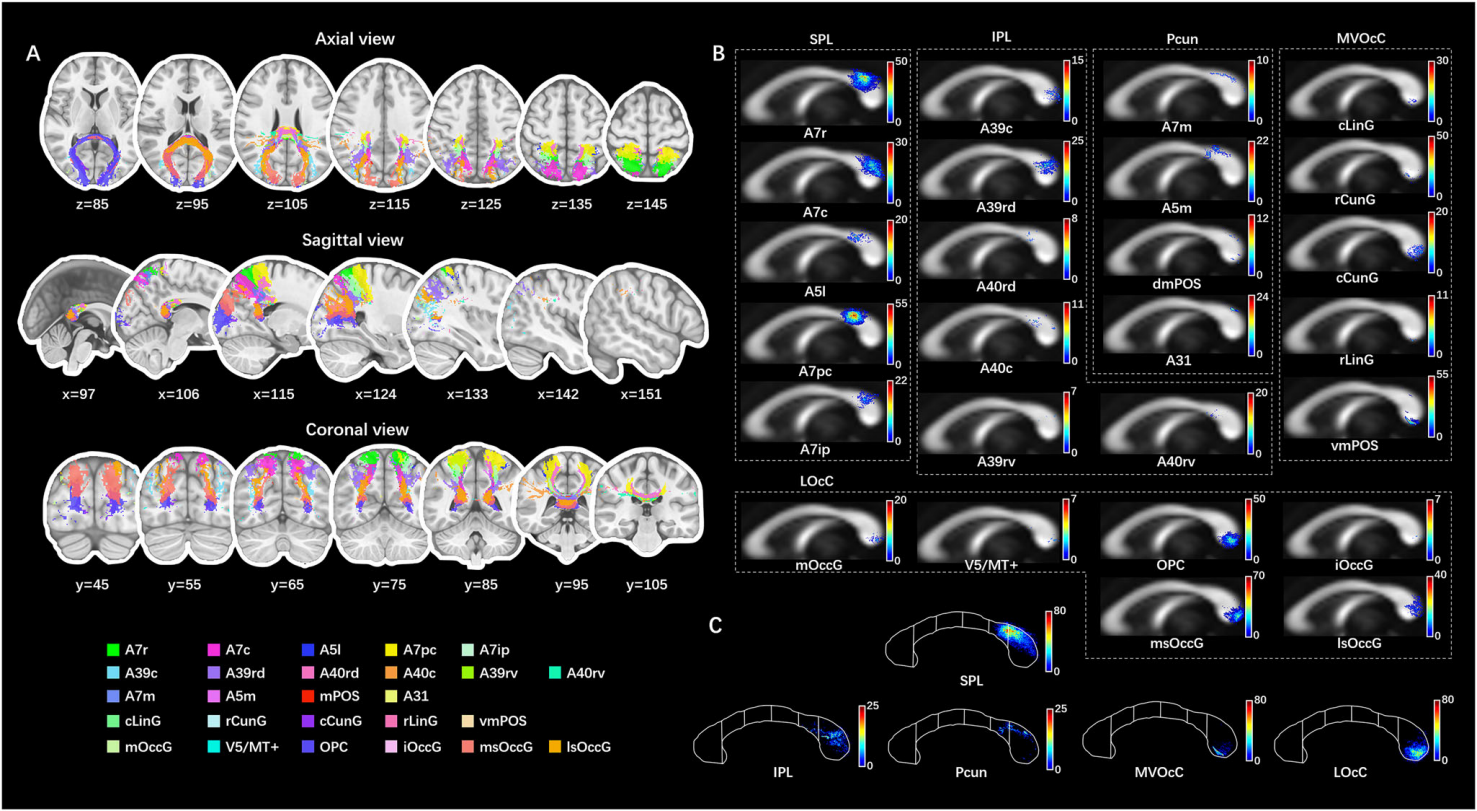
Trajectories and TDI of the parietal and occipital transcallosal tracts parcellated by the BNA. (A) Trajectories of the parietal and occipital transcallosal tracts parcellated based on the BNA were shown in two‐dimensional T1 sequences in MNI space. The parietal lobe contained 5 subregions in the SPL, 6 subregions in the IPL, and 4 subregions in the Pcun, while the occipital lobe included 5 subregions in the MVOcC and 6 subregions in the LOcC. (B and C) The fibers in TDI form were plotted at the callosal midsection. The subregions were named following the BNA's descriptions, which adopted the label schemes in the Brodmann atlas. (C) The subdivisions of the CC were segmented based on Witelson's scheme (Witelson 1989). The corresponding subregions of the rostral and caudal area 7, which presented predominant inter‐hemispheric connectivity in the parietal cortex, were orderly placed from the isthmus to dorsal splenium of CC; regarding the occipital cortices, the transcallosal fibers were generally distributed in the ventral splenium of CC. A31, area 31 (Lc1); A39c, caudal area 39 (PGp); A39rd, rostrodorsal area 39 (Hip3); A39rv, rostroventral area 39 (PGa); A40c, caudal area 40 (PFm); A40rd, rostrodorsal area 40 (PFt); A40rv, rostroventral area 40 (PFop); A5l, lateral area 5; A5m, medial area 5 (PEm); A7c, caudal area 7; A7ip, intraparietal area 7 (hIP3); A7m, medial area 7 (PEp); A7pc, postcentral area 7; A7r, rostral area 7; BNA, Human Brainnetome Atlas; cCunG, caudal cuneus gyrus; cLinG, caudal lingual gyrus; CC, corpus callosum; dmPOS, dorsomedial parietooccipital sulcus (PEr); iOccG, inferior occipital gyrus; IPL, inferior parietal lobule; LOcC, lateral occipital cortex; lsOccG, lateral superior occipital gyrus; mOccG, middle occipital gyrus; msOccG, medial superior occipital gyrus; MVOcC, medioventral occipital cortex; OPC, occipital polar cortex; Pcun, precuneus; rCunG, rostral cuneus gyrus; rLinG, rostral lingual gyrus; SPL, superior parietal lobule; TDI, track‐density image; V5/MT+, area V5/MT+; vmPOS, ventromedial parietooccipital sulcus.

Using high‐quality diffusion MRI data from 44 healthy individuals, homologous transcallosal tracts were reconstructed, including 41 macro‐anatomic and 101 function‐based parcellations. The corresponding trajectory and tract densities were mapped onto the callosal midsection, as shown in Figures 2, 3, 4, 5, 6. Additionally, in the connectome analysis, we observed heterogeneity in both the amounts and NQA values of the transcallosal tracts for the two atlases. The templates of transcallosal tracts, as well as callosal subregions, were created and made available for free at https://github.com/ousiqi/CCtemplate.

### 3.1 Callosal Templates Based on Anatomical and Functional Connectivity Parcellations

In the present study, the ICC of the segmentation of CC was 0.839 (95% confidential interval: from 0.508 to 0.933), while the overlap rate was 91.73% ± 3.54%, showing a relatively high reliability of our segmentation protocol. The template based on the AAL3 was composed of 41 different transcallosal commissural fibers as well as 41 corresponding callosal subregions, which corresponded to 17 frontal cortical areas, 6 occipital areas, 6 parietal areas, 8 temporal areas, 3 cingulate areas, and 1 insular area. The trajectory of fibers is shown in Figure 2. The callosal subregions were mapped onto the callosal midsection as a topography of in‐region fiber density, as shown in Figure 3. In general, the callosal tracts of the frontal lobes were distributed from the callosal rostrum to the posterior midbody, occupying the largest part of the CC (Figures 2A and 3C). The correspondence between the commissural fibers of other lobes and callosal subregions was as follows: (1) parietal lobe: callosal isthmus and rostro‐dorsal splenium (Figures 2B and 3D); (2) occipital lobe: caudo‐ventral splenium (Figures 2B and 3E); (3) temporal lobe: callosal rostrum and central splenium (Figures 2C and 3F); (4) insula, Rolandic operculum, and cingulate cortex: distributed ventrally and dorsally along the long axis of the CC (Figures 2D and 3G,H). On the callosal midsagittal section, we observed that fibers were densely distributed at the dorsal genu, rostral body, dorsal anterior midbody, posterior midbody, isthmus, and ventrocaudal splenium of the CC (Figure 3A,B).

Similar to the cortico‐callosal correspondence in the AAL3 group, the callosal subdivisions in the template based on the BNA constituted a more specific topography (Figures 4–6, Figure S1). In total, 101 transcallosal fibers of functionally homologous cortical regions, including 34 frontal (PFC region and central region shown in Figures 4 and 5, respectively), 19 parietal, 11 occipital (shown in Figure 6), 27 temporal, 3 insular, and 7 cingulate commissural fibers, were ultimately visualized and mapped on the CC. The subdivisions of the M1 are shown in Figure S1. However, the connections of the caudo‐posterior superior temporal sulcus and the dorsal agranular, granular, and dysgranular parts in the insula could not be reconstructed.

The results of the robustness analysis are provided in Section **S2.1**. The details of the fiber tracking and the two corresponding callosal topographies are described in Sections **S2.2 and S2.3**, respectively.

Consequently, the templates, including transcallosal tracts and topographies of the callosal subregions, comprise 41 and 101 parcellations based on the AAL3 and BNA, respectively.

### 3.2 Predominant Cortical Regions and Heterogeneous Callosal Tracts in ITN

**FIGURE 7:**
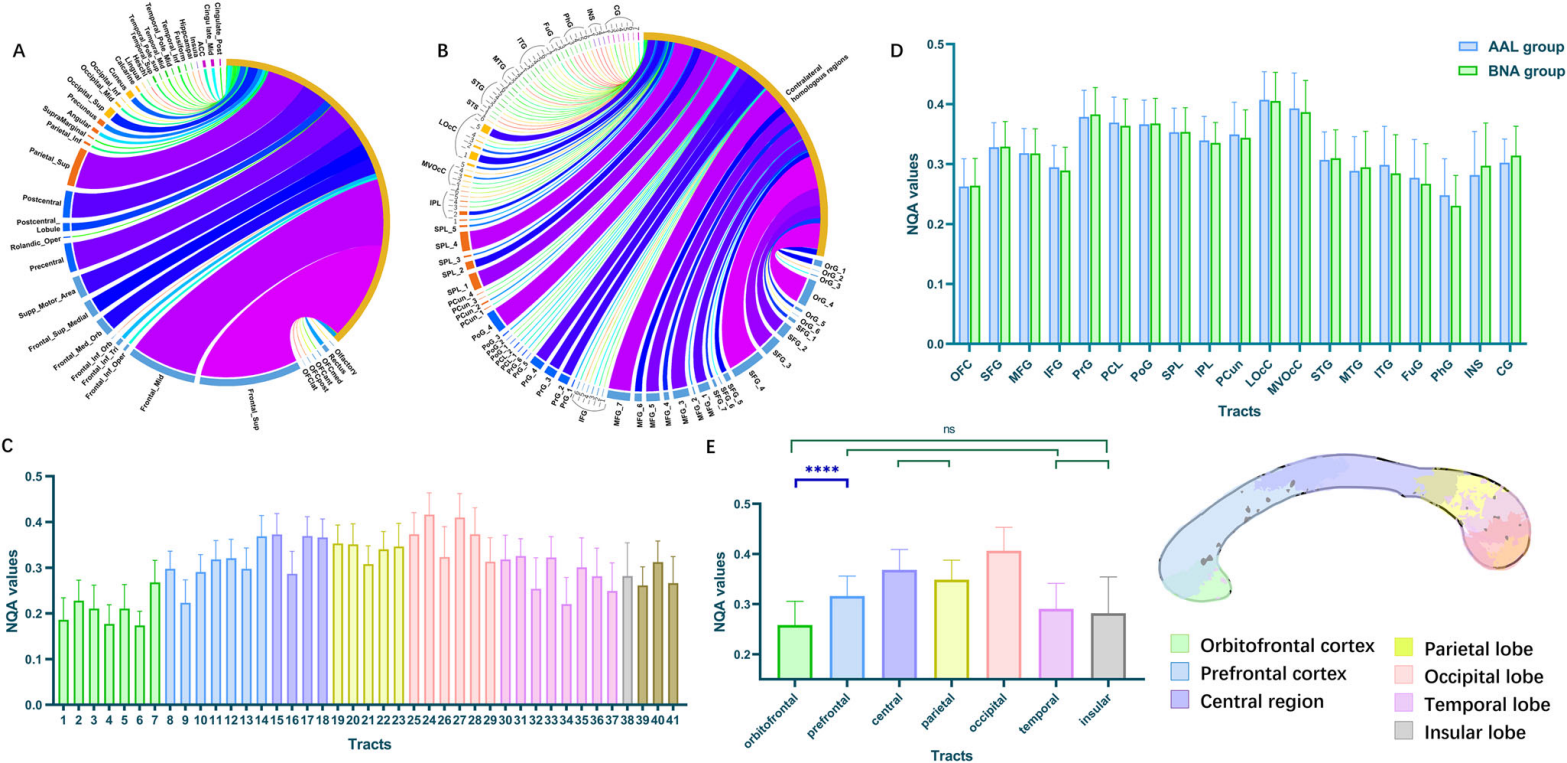
The connectome analysis of the interhemispheric transcallosal connectivity and the connectivity gradient of the callosal tracts. (A and B) The interhemispheric transcallosal connectivity was calculated based on the number of streamlines of different cortical subregions that were parcellated by the AAL3 and BNA, respectively. The list of abbreviations in (B) was provided in Table S3. (C) The level of the average NQA values of the transcallosal tracts connecting 41 cortical subregions in the AAL3 group was significantly heterogeneous. (D) The 41 and 101 groups of transcallosal fibers were re‐parcellated into 19 paired main bundles for comparison between the AAL3 and BNA groups. The results of paired *t*‐test and Wilcoxon test indicated the significant comparability between them, which suggested a stable difference and a gradient distribution of the connectivity of various callosal fibers. (E) The tracts were merged into seven bundles, corresponding to seven cortical subregions, to delineate the gradient of callosal connectivity based on the NQA values (left). We divided it into three degrees according to the ANOVA results: (1) the occipital visual cortex, (2) the central region and parietal cortex, (3) the PFC, temporal lobe, insular lobe, and OFC. It should be noted that, though in the same degree, the NQA values of the PFC and OFC were statistically different (ns: nonsense; ****p<0.0001). The corresponding subregions in the CC were displayed in different colors (right). Abbreviations: AAL3, Automated Anatomical Labeling atlas 3; BNA, Human Brainnetome Atlas; NQA, normalized quantitative anisotropy; PFC, prefrontal cortex; OFC, orbitofrontal cortex; CC, corpus callosum. 1, olfactory cortex; 2, gyrus rectus; 3, anterior orbital gyrus; 4, lateral orbital gyrus; 5, medial orbital gyrus; 6, posterior orbital gyrus; 7, medial orbital part of the superior frontal gyrus; 8, opercular part of the inferior frontal gyrus,; 9, pars orbitalis part of the inferior frontal gyrus; 10, triangular part of the inferior frontal gyrus; 11, middle frontal gyrus; 12, dorsolateral superior frontal gyrus; 13, medial superior frontal gyrus; 14, supplementary motor area; 15, precentral gyrus; 16, Rolandic operculum; 17, paracentral lobule; 18, postcentral gyrus; 19, superior parietal gyrus; 20, inferior parietal gyrus, excluding supramarginal and angular gyri; 21, supramarginal gyrus; 22, angular gyrus; 23, precuneus; 24, superior occipital gyrus; 25, middle occipital gyrus; 26, inferior occipital gyrus; 27, cuneus; 28, calcarine fissure and surrounding cortex; 29, lingual gyrus; 30, Heschl's gyrus; 31, superior temporal gyrus; 32, temporal pole of the superior temporal gyrus; 33, middle temporal gyrus; 34, temporal pole of the middle temporal gyrus; 35, inferior temporal gyrus; 36, fusiform gyrus; 37, parahippocampal gyrus; 38, insula; 39, anterior cingulate & paracingulate gyri; 40, middle cingulate & paracingulate gyri; 41, posterior cingulate gyrus.

Cortical regions with rich ITC were identified in the connectome analysis of streamline number. In the AAL3 group, a total of up to 92.5% of the callosal fibers were connected to these regions, including the dorsolateral PFC (DLPFC), medial PFC (MPFC), supplementary motor area (SMA), primary somatosensory cortex (M1), primary motor cortex (S1), superior parietal lobule (SPL), and occipital cortex, showing a predominant richness of connections. Their respective proportions are as follows: 45.6%, 9.6%, 5.6%, 9.6%, 6.9%, 10.1%, and 5.2% (shown in Figure 7A and Table S2). For the BNA group, these predominant connections were more specifically refined (Figure 7B and Table S3). Among them, connections with proportions above 2% of the total number of streamlines included the following: medial area 11 in the OFC; dorsolateral areas 6, 8, and 9 in the superior frontal gyrus (SFG); area 46, ventrolateral areas 8 and 6, lateral area 10 in the middle frontal gyrus (MFG); caudal dorsolateral area 6, area 4 in the precentral gyrus (PrG); areas 1–3 in the trunk region of the postcentral gyrus (PoG); area 7 in the SPL; occipital polar cortex and medial superior occipital areas.

NQA values were measured to identify callosal tracts with different diffusion characteristics and connectivity. The NQA values of the 41 transcallosal tracts in the AAL3 group were heterogeneous (Kruskal–Wallis nonparametric test: *H* = 1015.897, *p* < 0.0001). Callosal fibers with the highest NQA values were found mainly connecting the occipital, M1, S1, and parietal areas (Figure 7C and Table S2), followed by tracts connecting temporal and prefrontal areas. The lowest values were observed for the temporal pole and OFC. In the BNA group, the 20 tracts with highest NQA values mainly connected the parcellations of the M1 (trunk and upper and lower limb regions of area 4), SMA (area 6), S1 (trunk and lower limb region of areas 1–3), lateral occipital areas, parietal cortex (areas 5 and 7), and posterior superior temporal sulcus (Table S3). Further analyses revealed no significant differences among the 19 pairs of re‐parcellated bundles (paired *t*‐tests for six pairs and Wilcoxon tests for the other 13, *p* > 0.5; as shown in Figure 7D and Table S4. Among these bundles, high repeatability was observed in 15 pairs (ICC values > 0.75), while the repeatability of the others, including the precuneus, medioventral occipital cortex, parahippocampal gyrus, and cingulate gyrus, was moderate (ICC values, 0.488–0.748).

Last, among the seven cortical subregions, the PFC exhibited the richest ITC according to the proportion of streamlines (56.61% in AAL3 and 46.81% in BNA), followed by the central region (17.43% in AAL3 and 18.09% in BNA), parietal lobe (13.41% in AAL3 and 16.49% in BNA), OFC (5.78% in AAL3 and 11.00% in BNA), and occipital lobe (5.20% in AAL3 and 6.69% in BNA). In terms of the bundles connecting these regions, significant heterogeneity of NQA values was also observed (*F* (6133.40) = 51.72, *p* < 0.001). In descending order, mean NQA values for the connecting tracts were ranked as follows: occipital lobe (0.406 ± 0.047), central region (0.368 ± 0.041), parietal lobe (0.349 ± 0.039), PFC (0.316 ± 0.040), temporal lobe (0.291 ± 0.051), insular lobe (0.282 ± 0.073), and OFC (0.258 ± 0.047) (Figure 7E and Table S5). Based on the results of post hoc tests (Table S6), we further divided the transcallosal tracts of the seven subregions into three gradients: (1) the apex of the gradient for the occipital lobe; (2) the central and parietal lobes; and (3) the PFC, temporal lobe, insular lobe, and the end of the OFC. It should be noted that, though divided in the same gradient, NQA values for the tracts corresponding to the PFC were still higher than those for the OFC (mean difference: 0.058 ± 0.011, *p* < 0.0001). Accordingly, fibers in different callosal subregions were also divided into three gradients of connectivity: (1) the ventro‐caudal splenium; (2) the midbody, containing the region from the isthmus to the dorso‐rostral splenium; (3) the region containing the dorsal genu, rostral body to anterior midbody, and central splenium, as well as the area from the ventral genu to the rostrum of the CC (Figure 7E).

## 4 Discussion

This study utilizes multiple directions and *b* values for QA‐based DSI tractography of the CC, which allowed us to develop a callosal template, including the homologous transcallosal tracts and topographies of callosal subregions, corresponding to 41 anatomical and 101 functional cortical parcellations, respectively. Additionally, we identified significant heterogeneity in the ITC of homologous cortical regions and the connectivity patterns of callosal fibers. This heterogeneity, potentially influenced by the microarchitecture of callosal fibers and the functional gradients of cortical organization established during development, underscores the CC's diverse roles in integrating cortical functions. The template, highlighting these structural and functional complexities, is freely available for potential clinical applications and brain research.

### 4.1 Quantitative Anisotropy Reflects the Relationship Between Patterns of Interhemispheric Connectivity and Cortical Development

In this study, heterogeneity of callosal connectivity existed in terms of the number of streamlines and diffusion scalars in the two different parcellation schemes. The observed heterogeneity in callosal connectivity provides crucial insights into the hierarchical organization of brain networks. Based on quantitative fiber‐tracking, predominant homologous cortical regions with richer ITC were reflected by a higher proportion of transcallosal streamlines (Yeh et al. 2021). Thus, they can be classified into two types of closely connected regions. On the one hand, the PFC and OFC (especially the medial OFC, DLPFC, and MPFC) which are involved in motor planning (Schilling et al. 2013), self‐evaluation (Beer et al. 2010), and reward circuitry (Wan et al. 2020), accounted for more than half of streamlines in our study (62.39% in AAL3 and 57.81% in BNA). This may be related to the disproportional expansion of the human frontal association network when compared with that in non‐human primates (Catani 2019; Fjell et al. 2015). On the other hand, rich ITC was observed in the sensorimotor cortices (including the motor, somatosensory, and occipital vision areas) and the parietal cortices, which exhibit functions related to multisensory integration (e.g., audiovisual (Molholm et al. 2006) and visuospatial processing (Nachev and Husain 2006)). Their callosal fibers accounted for 36.04% in the AAL3 analyses and 41.27% in the BNA analyses. As in a previous study (Zarei et al. 2006), except for the unimodal sensorimotor cortices, our results highlighted the functions of subregional modules such as vision‐action and attention in the interhemispheric integration of information in BA7 of the SPL (Wang et al. 2015). Thus, the current results may reflect a bifurcate pattern of ITC of cortical regions from low‐order to high‐order functions (Friedrich, Forkel et al. 2020; Friedrich, Fraenz, et al. 2020).

We also observed heterogeneity in QA values measured along the callosal tracts (Figure 7E). Our results echoed those of studies on the principal gradient of cortical organization (Margulies et al. 2016), indicating that the hierarchical gradient of functional processing mapped onto the CC (Friedrich, Forkel et al. 2020) was inversely correlated with the connectivity gradient of the callosal tracts delineated using QA values. Since the QA values reflect the diffusion characteristics of fibers, this result may imply a correspondence between the hierarchical gradient of cortical function and the connectivity determined by the microarchitecture of the callosal fibers. First, from the perspective of structural connectomics, the gradient of QA values for these callosal fibers verified the diverse microarchitectural characteristics observed in the histological research of Aboitiz et al., such as the diameter and thickness of the myelin sheath (Aboitiz and Montiel 2003; Aboitiz et al. 1992). Additional research has demonstrated that these characteristics determine the axonal conduction velocity (ACV) of interhemispheric communication (Caminiti et al. 2013). Hence, by reflecting the communicating velocity, the QA values observed in our study imply various connectivity gradients for callosal fibers of various cortical regions. According to the callosal topography we plotted, the callosal tracts of the occipital and motor/somatosensory cortices were mainly composed of large‐diameter fibers (diameter >3 µm) (Phillips et al. 2015), thus exhibiting a relatively higher ACV and connectivity gradient than others. In contrast, most orbitofrontal commissural fibers were thin (diameter <1 µm), corresponding to a low ACV and connectivity gradient. Further, the high connectivity gradient for callosal fibers of the sensorimotor cortices may provide complementary evidence regarding the anchoring role of these primary cortices in cortical ontogeny and phylogeny (Oligschläger et al. 2017). Recent research into the hierarchical gradient of functional processing suggests that the commissural fibers in the ventral splenium, posterior midbody, and isthmus are projections from lower‐level sensorimotor areas that peak in the negative spectrum (Friedrich, Forkel et al. 2020; Margulies et al. 2016). These unimodal areas are considered as possible core organizing fields that act as anchors in the development of the cerebral cortex (Buckner and Krienen 2013). Thus, they may be fundamental for interhemispheric communication, which may be reflected in their superior connectivity gradient (Innocenti et al. 2022). In contrast, the fibers in the rostrum and genu mainly connect to higher‐level multimodal areas related to the associative and default mode networks, such as the temporal cortex, MPFC, and OFC. These areas may emerge later in development (Buckner and Krienen 2013) and thus possess an inferior gradient.

Accordingly, the above evidence concerning the ITC of homologous cortical regions and the connectivity of callosal fibers may converge toward the conclusion that heterogeneity in callosal connectivity is representative of evolutionary stability (Friedrich, Forkel et al. 2020), with support from both non‐human primate (Oligschläger et al. 2019) and human (Oligschläger et al. 2017) studies that discovered a similar connectivity gradient and patterns of expansion (Hill et al. 2010). Other studies support the notion that functional patterns are related to the intrinsic geometry of the cortex formed during evolution (Huntenburg et al. 2018). Therefore, our quantitative results concerning the connectivity of homologous cortical regions and corresponding callosal tracts are mutually enriching, complementary, and integrative.

The heterogeneity we observed in callosal fiber density aligns with recent whole‐brain white matter atlas findings. Radwan et al. (2022) demonstrated that bundle density is a key determinant of tractography reliability—dense bundles like the pyramidal tracts show high reproducibility, while sparser bundles such as the fornix exhibit greater variability. Our observation that prefrontal and temporal callosal fibers were relatively sparse and more challenging to reconstruct is consistent with this principle, highlighting how fine‐grained analysis of a single structure can both build upon and contextualize whole‐brain mapping efforts.

### 4.2 A Callosal Template Incorporating Anatomical and Functional Connectivity

In this study, we developed a callosal template reflecting both cortical macro‐anatomic labels and long‐range functional connectivity, which has not been reported in previous studies. Previous studies focused on global connectivity (Styner et al. 2005; Wang et al. 2020, 2021), more precise cortical segmentations (Archer et al. 2018; De Benedictis et al. 2016; Domin and Lotze 2019), the relationship between callosal microstructure and diffusion characteristics (Björnholm et al. 2017; Caminiti et al. 2013; Friedrich, Fraenz, et al. 2020; Lee et al. 2019; Liu et al. 2010; Park et al. 2011; Pizzini et al. 2009), or cortico‐callosal patterns based on RSFC (Friedrich, Forkel et al. 2020; Wang et al. 2021). However, no previous studies have generated a callosal template for precise clinical and surgical evaluation that combines patterns of anatomical and functional connectivity.

In terms of cortical functional subregions, our template followed the fundamental concept that only one critical determinant of regional specialization is represented in interhemispheric long‐range connectivity (Fan et al. 2016). Based on this concept, the transcallosal fibers of the subdivisions in M1 differ substantially (Domin and Lotze 2019). In our study, we included trunk‐represented, and the resulting callosal subregions mapped based on M1–M1 connections also showed precise differentiation that largely agreed with previous work (Domin and Lotze 2019) (Figure S1). Further extending from M1 to global homologous subregions, substantial differentiation was still observed in both the transcallosal tracts and corresponding CC subregions. Thus, our template can provide rich and sophisticated details, given that nearly all cortical functional subdivisions were mapped. Furthermore, our study included more callosal subregions based on cortical anatomical labels than previous studies (Archer et al. 2019; Pannek et al. 2010). Analyses of these callosal subregions, such as the prefrontal‐mapped regions (medial SFG, SFG, MFG, and inferior frontal gyrus) revealed that our results were more in line with the dorsal‐ventral and laminate‐formed arrangement that was observed in post‐mortem neuroanatomical experiments (De Benedictis et al. 2016). We attributed this finding to the acquisition scheme, which included 258 directions and multiple *b* values (maximum reaching 7000 s/mm^2^). Such schemes have been considered more appropriate for connectomic studies than single‐shell schemes, for example, DTI and HARDI (Yeh and Verstynen 2016). As high *b* values can significantly improve the reproducibility and accuracy of fiber tracking (Pannek et al. 2010), QA‐based deterministic tractography allows for more precise assessment of complex patterns of fiber crossing, thus making our template more reliable (Park et al. 2008; Yeh et al. 2013). The critical role of such advanced diffusion models is further underscored by recent whole‐brain atlasing efforts. For instance, Radwan et al. (2022) employed probabilistic CSD tractography to map 68 major fasciculi, providing a comprehensive normative reference for major white matter pathways. While their work offers a broad overview, our study complements this by providing a high‐resolution, functionally‐informed parcellation of a single critical structure—the corpus callosum. Together, these approaches underscore the importance of multi‐scale white matter mapping: whole‐brain atlases provide essential context and normative references, while structure‐specific templates enable detailed investigation of regional heterogeneity and support precise clinical applications.

Although the gyri in the BNA were parcellated based on the Desikan–Killiany Atlas (Desikan et al. 2006), which is slightly different from the AAL3, both atlases used for the current study subdivide the cerebral cortex into gyri‐based regions, resulting in comparable neuroanatomical division. Consequently, cortico‐callosal mapping based on the BNA and AAL3 can provide important complementary information regarding interhemispheric long‐range connectivity based on macro‐anatomic labels.

### 4.3 A Practical Callosal Template for Clinical Application

Our callosal template can accurately reflect the diverse microarchitectural characteristics of callosal fibers as well as the natural connectivity patterns determined by the cortical functional gradient formed in development. Combining cortical parcellations based on anatomical and functional connectivity may help to improve understanding of the relationships between structure and functional connectivity in ITN. Through image registration based on MNI space, our template may become useful in clinical applications for individual patients, as it can be used to accurately define the anatomical position of callosal fibers that correspond to given cortical subregions (or vice versa) based on simple T1 three‐dimensional images from the patient. Hence, our template may enable more specific measurements of pathological changes in the microstructure of the transcallosal tracts (Ouyang et al. 2015) and precise evaluation of neurological diseases involving the CC (e.g., infarction (Sun et al. 2019) and epilepsy (Chen et al. 2020)), which can in turn guide more accurate treatment. Further, our template may be applicable for preoperative evaluation and surgical planning for patients undergoing thermal therapy for callosal tumors (Beaumont et al. 2018), precise mini‐invasive callosal callosotomy (Roland et al. 2019), or even prospective neuromodulation in cases of refractory epilepsy (Couturier and Durand 2018), in which precise positioning is crucial to avoid damage to callosal fibers and preserve normal function.

Given that current methods cannot achieve perfectly clear, non‐overlapping callosal segmentation, we recommend using this template as a probabilistic reference framework rather than rigid anatomical boundaries. Researchers should apply a probability threshold (e.g., >50%) to identify consistent fiber distributions. For surgical planning, the template should complement—not replace—individual patient tractography. The template is best suited for group‐level comparisons and hypothesis generation, and should not be used for precise subject‐specific claims in regions with substantial fiber overlap (e.g., the splenium).

### 4.4 Limitations

Our study had some limitations. First, the transcallosal streamlines connecting temporal and lateral orbitofrontal regions were relatively low in number when compared with those connecting other regions, while a “gap” with relatively sparse fibers connecting prefrontal and temporal areas was observed in the rostro‐ventral splenium. These fibers were also poorly reconstructed in comparison, possibly because the relevant regions are located furthest from the CC, including the fibers connecting both prefrontal areas passing through the splenium. Additionally, we selected the optimal QA threshold to avoid tracking false‐positive fibers, although the threshold may have been higher than part of the true fibers. In future studies, acquisition schemes with higher *b* values can be used to address this issue, although they did not seem to have a large influence on our current results. Second, we did not quantitatively investigate the relationship between historically myeloarchitectural characteristics in callosal areas (tract density, diameter, etc.) and QA values for the callosal tracts. Further studies are required to clarify this issue and complement the current findings. Third, areas with relatively sparse tract distribution may seem inconspicuous in visual analysis based on the super‐resolution TDI format, as the locally concentrated parts raise the maximum value overall. Increasing image resolution may help to address this issue. Fourth, while our sample of 44 participants is within the range of many classic tractography atlases, we acknowledge that recent large‐scale studies have included approximately 100 participants. Therefore, our sample size remains modest, and the generalizability of our findings, particularly for fiber bundles with higher variability, may be enhanced by future studies with larger, more diverse cohorts. Further, the limitations of this study include the narrow age range (18–30 years) and the unequal sex distribution (16 males and 28 females) in the dataset. While the selected cohort provides valuable insights into the callosal connectivity in young adults, these demographic constraints may limit the generalizability of our findings to broader populations. Future studies should incorporate a wider age range and achieve a more balanced sex distribution to ensure the atlas's applicability across diverse demographics and to explore potential developmental and sex‐based differences in callosal connectivity. Last, in addition to evidence from diffusion MRI, studies should attempt to analyze actual callosal myeloarchitectural features and perform electrophysiological experiments for further analysis of transcallosal connections.

## 5 Conclusion

Our study has developed a callosal template incorporating macro‐anatomic and long‐range connectional information, providing detailed transcallosal tracts and the corresponding topography of callosal subregions at high resolution using DSI techniques. In addition, the QA‐based structural connectome observed in this study reflects the relationship between patterns of interhemispheric connectivity and cortical development. This template will be freely available for download and may aid researchers in evaluating and treating neurological diseases involving the CC and its connections to cortical areas.

## Author Contributions

Conceptualization: Guoguang Zhao and Jie Lu. Methodology, software, and visualization: Siqi Ou, Chao Zhang, Jianwei Shi, and Penghu Wei. Validation: Siqi Ou and Zhenming Wang. Investigation: Chao Zhang, Jianwei Shi, Zhenming Wang, Penghu Wei, Siqi Ou, Chao Lu, Yihe Wang, Huaqiang Zhang, and Xiaotong Fan. Formal Analysis: Chao Zhang, Jianwei Shi, Siqi Ou, and Penghu Wei. Data curation: Chao Zhang, Zhenming Wang, and Siqi Ou. Writing – original draft: all authors. Writing – review and editing: all authors. Supervision: Guoguang Zhao, Jie Lu, and Yongzhi Shan. Project administration: Guoguang Zhao. Funding acquisition: Guoguang Zhao.

## Funding

This study was supported by the National Natural Science Foundation of China (Grant Numbers 81871009, 81801288, 82030037).

## Ethics Approval Statement

The authors confirm that we have read the Journal's position on issues involved in ethical publication and affirm that this report is consistent with those guidelines.

## Conflicts of Interest

All authors declare that they have no conflicts of interest.

## Supporting information




**Supplementary Materials**: brb371306‐sup‐0001‐SuppMat.docx

**Supplementary FigureS1**: brb371306‐sup‐0002‐FigureS1.tif

## Data Availability

Anonymized data used for this study will be made available from the corresponding author upon reasonable request. The templates of transcallosal tracts, as well as callosal subregions, were created and made available for free at https://github.com/ousiqi/CCtemplate.
