# Supplementary material for: Mapping Human Corpus Callosum Connectivity With Diffusion Spectrum Imaging: A Deterministic Tractography Approach
Source: Brain Behav. 2026 Mar 12;16(3):e71306. doi: 10.1002/brb3.71306 (PMC13093777; doi:10.1002/brb3.71306)
Supplement: Supplementary file 1 — Supplementary Materials: brb371306‐sup‐0001‐SuppMat.docx [file BRB3-16-e71306-s002.docx]

**1. Material and Methods**

**1.1 The recruitment**

Our participant pool included healthy individuals born in China. Staffed by our recruiting efforts, the young social community and university students were initially screened over the telephone to confirm that they met the inclusion criteria and were willing to participant in our experiment. The inclusion criteria were as follows: (1) aged 18–30 years; (2) without neurological or psychotropic disorders; and (3) without contraindication to magnetic resonance imaging (MRI) examination. Finally, 44 young healthy volunteers (aged 18–30 years, average age 24.0 years; 16 men and 28 women) passed the screening with preclusion of neurological or psychotropic disorders and recruited in our study.

- 1. **MRI scanning parameters and quality control procedures**

**(1) MRI scanning parameters**

The MRI data were acquired using a GE Signa Premier 3.0T scanner (General Electric Healthcare, Waukesha, WI, USA) at Xuanwu Hospital. The half-q-space diffusion spectrum imaging (DSI) data were acquired using a 64-channel head coil (258 directions). The DSI scanning parameters were as follows: b values, 0–7000 s/mm2; repetition time (TR)/echo time (TE), 5548 ms/84.1 ms; field of view (FOV), 112 mm × 112 mm; voxel size, 2 mm × 2 mm × 2 mm; flip angle, 90°; and acquisition time, 26 minutes. The parameters for three-dimensional T1 (magnetization-prepared rapid acquisition gradient echo sequence) structural images were as follows: TR/TE, 2420 ms/2.376 ms; FOV, 256 mm × 256 mm; voxel size, 1 mm × 1 mm × 1 mm; and acquisition time, 6 minutes.

1. **Data preprocessing**

All raw diffusion data were imported into DSI-Studio software ([http://dsi-studio.labsolver.org](http://dsi-studio.labsolver.org/)) for preprocessing and reconstruction. Q-space diffeomorphic reconstruction (QSDR), a model-free reconstruction technique, was employed to reconstruct individual spin distribution functions (SDFs) and simultaneously normalize them to the Montreal Neurological Institute (MNI) space (ICBM-152 template). This approach enables direct group-level comparisons without requiring separate spatial normalization steps. The reconstruction parameters were as follows: diffusion sampling length ratio, 1.2; SDF tessellation, 20-fold; and number of fibers resolved per voxel, 10. The registration quality between each warped image and the template was evaluated using the R² value, with R² > 60 considered indicative of successful registration. Then, 44 individual representations of normalized SDF maps and a group-averaged template in the QSDR space were acquired, and used for further fiber tracking.

1. **Quality control procedures**

Quality control of data acquisition and preprocessing was independently performed by two experienced radiologists following a standardized protocol comprising sequential steps: 1) Real-time inspection during acquisition for obvious artifacts (e.g., head motion, signal dropout), with failed data re-acquired immediately; 2) Post-acquisition visual inspection of all raw images by both radiologists; 3) Quantitative assessment of average QA values during preprocessing, with abnormally low values flagged for further scrutiny; 4) Verification of image orientation and file integrity, excluding datasets with flipped orientation, corrupted files, or incomplete b-vector information; and 5) Inter-rater consensus to resolve any discrepancies through discussion.

Based on these procedures, 46 participants were initially recruited. Two were excluded: one due to excessive head motion (confirmed by low QA values and visible ghosting artifacts) and one due to image artifacts (signal dropout). The remaining 44 participants met all quality criteria and were included in the final analysis.

- 1. **Defining the regions of interest (ROI)**

Since the quantitative anisotropy (QA) space is reconstructed based on diffusion data, which is vulnerable to spatial warping during data acquisition compared to structural images (e.g., T1 and T2), directly applying a segmentation of the corpus callosum (CC) based on structural images may cause undesired spatial bias in the QA space owing to different trends in warping. As fiber tracking would ultimately be performed in the QA space, we decided to segment the CC on the QA map. According to the boundary of intensities, the ROI of the CC was outlined manually by two raters on the mid-sagittal QA sequences and one more layer in the horizontal direction. Because the slice thickness was 2 mm, the overall thickness of the plane was 6 mm. This procedure was conducted using the same protocol for all 44 participants. Further, we employed the intraclass correlation coefficient (ICC) for absolute agreement to quantify the consistency between two raters regarding the volume of the ROI and the voxel overlap rate on the QA map, thereby assessing the reliability of the ROI definition. The overlap rate was defined as the percentage of the overlapped region compared with the segmentation of the first rater. The area overlapped between the two raters was used as the ROI for fiber tracking.

- 1. **Parameters in fiber tracking**

We performed deterministic tractography with DSI-Studio software using the following settings: step size, 0.5 mm; smoothing, 0.8; angular threshold, 90; minimum length, 5.0 mm; maximum length, 300 mm; and the tracking process was set to terminate if the seed number reached 50,000.

- 1. **Resampling and visualization of the tracts**

To improve the visual resolution, image interpolation of the MNI152-T1 space and averaged QA space that resampled the voxel to 0.5 mm were conducted with MATLAB (MathWorks, Inc., Natick, MA). The codes were provided in Supplemental 1.8. The regions of pathways in different given colors were overlaid on the resampled T1 images, while the super-resolution track-density image (sr-TDI) was displayed in the indigo-crimson temperature map on the averaged QA images, respectively. The aforementioned data were projected to the MNI space and ultimately presented using ITK-SNAP 3.8.0 software.

- 1. **Re-parcellation of both atlases**

The global cortical regions of both hemispheres were re-parcellated into 19 structures with obvious macro-anatomic features, which were paired between the AAL3 and BNA. These cortical regions of each hemisphere included 4 prefrontal areas (the orbitofrontal cortex [OFC], superior frontal gyrus [SFG], middle frontal gyrus [MFG], and inferior frontal gyrus [IFG]), 3 central cortical areas (the precentral gyrus [PrG], postcentral gyrus [PoG], and paracentral gyrus), 3 parietal areas (the superior parietal lobe [SPL], inferior parietal lobe, and precuneus), 2 occipital areas (medioventral occipital cortex [MVOcC] and lateral occipital cortex [LOcC]), 5 temporal areas (the superior temporal gyrus, middle temporal gyrus, inferior temporal gyrus, fusiform gyrus, and parahippocampal gyrus), insular lobe, and cingulate gyrus. The components of these 19 cortical regions in each group were shown in Table S4.

- 1. **Creation of the templates**

We first projected the standard T1 sequence of MNI152 atlas to the group averaged template in QA space. Then, the callosal fibers with lengths of 4 mm on the left and right sides of the plane including the callosal midsection were selected for further analysis. In total, 41 callosal subdivisions corresponding to the AAL3 and 101 subdivisions corresponding to the BNA were plotted on the CC, which could be regarded as two callosal topographies. Both of these transcallosal tracts and callosal topographies were exported to “region” formats and registered to T1 sequence of MNI152 atlas using the statistical parametric mapping (SPM) toolbox. Subsequently, with self-written codes in MATLAB, we in-orderly set different image intensities for these fibers in the two atlases, respectively. Then they were transformed and integrated into 4D nifti format. Finally, the templates of transcallosal tracts and callosal topographies that parcellated according to the AAL3 and BNA were created.

- 1. **Data and code availability statement**

The statistical data were provided in Tables S1-5 in Supplementary Material. The MRI data of volunteers are not available to the public due to privacy issues and restrictions imposed by the Ethical Committee of Xuanwu Hospital. DSI Studio is available at <http://dsi-studio.labsolver.org>. The MNI152 atlas is available at <http://www.bic.mni.mcgill.ca/ServicesAtlases/ICBM152-NLin2009>. The MATLAB is available at <https://fr.mathworks.com>. The statistical parametric mapping (SPM) toolbox is available at <https://www.fil.ion.ucl.ac.uk/spm>. The ITK-SNAP 3.8.0 software is available at <http://www.itksnap.org/pmwiki/pmwiki.php>. The Automated Anatomical Labeling atlas 3 (AAL3) is available at <http://www.gin.cnrs.fr/tools/aal-aal>3, and the Human Brainnetome Atlas (BNA) is available at <http://atlas.brainnetome.org/download.html>. Circular graph is available at <http://mkweb.bcgsc.ca/tableviewer>.

The code for resampling (the voxel size of original QA images is 2 mm × 2 mm × 2 mm):

QA_orig=niftiread('QA.nii');

info=niftiinfo('QA.nii');

s=info.PixelDimensions;

v=info.ImageSize;

QA =imresize3(QA _orig, [2*s(1)*v(1), 2*s(2)*v(2), 2*s(3)*v(3)]);

info.ImageSize=size(QA);

info.PixelDimensions=[0.5,0.5,0.5];

niftiwrite(QA, 'QA _05mm.nii', info)

1. **Results**
   1. **Robustness analysis**

Using QSDR method, all 44 individual SDFs were normalized with good registration (R-squared value range, 64–76; Table S1). The transcallosal commissural ratios between the number of transcallosal tracts and global streamlines were in normal distribution (P = 0.628). There was no significant difference between the ratios in the 44 individual QA spaces and averaged QA template (*R_i_* = 0.823±0.038, *R_a_* = 0.831, P = 0.187), suggesting that the optimal QA thresholds determined individually were well representative (Table S1).

- 1. **Fiber tracking**

By tracking the fibers projected from the left or right cortices to the CC, respectively, the transcallosal fibers were visualized in all cases. After merging and eliminating the heterogeneous transcallosal fibers, most of the connections between both homologous cortical areas parcellated by AAL3 were almost reserved in all 44 participants except the fibers connecting the subregions that were close to the skull base, such as the OFC, inferior frontal and occipital areas. That was partly because the optimal QA threshold set in fiber tracking process was too high to track these fibers. Therefore, we adjusted the QA threshold downward appropriately only when tracking these fibers, and then we filtered out those false-positive connections carefully. Likewise, we managed these parcellations of the BNA group in this way for tracking as much proper fibers as possible. Ultimately, the majority of fibers connecting the BNA-parcellations were retained. The parcellations and fibers’ amounts were performed in Table S2-3. The trajectory and sr-TDI of all transcallosal fibers were presented in the resampled MNI-T1 and averaged QA templates, respectively.

- 1. **The transcallosal tracts template and callosal topography based on different parcellations**

For the transcallosal tracts template based on the AAL3 parcellations, it was composed of 41 different commissural tracts, which included 17 frontal cortical areas, six occipital areas, six parietal areas, eight temporal areas, three cingulate areas, and one insular area. The fibers pathways in the form of regions were shown in Figures 2A-D, while the fibers’ corresponding in-region density were mapped on the callosal midsection planes, shown in Figures 3C-H. For the callosal topography, the indigo-crimson temperature map represented the account of fibers traversing each sub-voxel on the topography of the CC (Figs. 3B-H). Higher tract density was indicative of more transcallosal connections corresponding to homologous cortical regions at the group level, which indicated that the majority of them passed through the dorsal genu, rostral body, dorsal anterior midbody, posterior midbody, isthmus, and ventrocaudal splenium of the CC (Figs. 3A-B). Since the track-density of different tracts was a continuous variable in this study, we displayed it with an appropriate range that the maximum of each colormap (the most crimson end) was defined as 30%-50% of the maximum of track-density in that area (Fig. 3B-H). Thus, the density of transcallosal tracts of each division on this map indicated the principle of the tracts’ distribution at a group level. From the perspective of specific regions, tracts connected to the frontal lobe were generally distributed from the rostrum to the posterior midbody of the CC (Fig. 3C). The orbitofrontal tracts mainly passed through the rostrum and ventral genu, while the fibers connecting the medial prefrontal cortex (MPFC) and dorsolateral prefrontal cortex (DLPFC) traversed the dorsal genu and rostral body. The predominant regions in which fibers related to the supplementary motor area (SMA) and PrG were mainly in the anterior midbody and posterior midbody, respectively. In the parietal lobe, the transcallosal fibers traveled across the isthmus and rostro-dorsal part of the splenium (Fig. 3D). The predominant subareas that were roughly located in the isthmus and rostro-dorsal splenium of the CC were transcallosal fibers corresponding to the PoG and SPL, respectively. Regarding the occipital lobes, including the medial and lateral cortical regions, the commissural fibers generally intersected the ventrocaudal splenium of the CC (Fig. 3E). The neural pathways connecting both temporal lobes were generally mainly distributed in the central part of the splenium, while a few of them connecting the temporal pole was in the rostrum (Fig. 3F). The fibers interconnected to both insular lobes distributed relatively extensively that they passed the genu, ventral part of the rostral body and anterior midbody (Fig. 3G). For the cingulate cortex, which was segmented into three parts from anteriorly to posteriorly, the corresponding transcallosal fibers were distributed in the order of a certain special position that was close to the inferior surface of the rostrum and genu, and the superior surface of the bulk of CC (Fig. 3H).

For the transcallosal tracts template based on the BNA parcellations, 101 transcallosal connections of functionally homogeneous cortical regions, including 34 frontal, 19 parietal, 11 occipital, 27 temporal, 3 insular and 7 cingulate commissural tracts were ultimately visualized based on the BNA parcellations (Figs. 4-5, S2). The connections of the caudoposterior superior temporal sulcus, the dorsal agranular, granular and dysgranular parts in insula were incapably reconstructed. In this part, the commissural tracts connected to the PrG, PoG, and paracentral lobes were presented separately to highlight the connectional segmentations of the central region (Fig. 5), including the primary motor cortex (M1) and somatosensory cortex (S1), while other regions in the frontal lobe were defined as the prefrontal areas (Fig. 4). Similar to the corresponding anatomic spatial relationship between the cortical subregions and CC in the AAL3, the subdivisions within different Brodmann areas based on the BNA constituted a more specific topography of the CC (Fig. 4-6). In the PFC, the predominant subregions having a relatively higher density and larger quantity of transcallosal fibers, mainly located at DLPFC (dorsolateral area 6, lateral area 8, dorsal area 9/46) and the frontal pole (FP) (lateral area 10 and medial area 11) (Fig. 4). For the central region, the relatively predominant segmentations were caudal dorsolateral area 6, upper limb and trunk region of area 4, and trunk region of area 1-3 (Fig. 5). We also noted that the transcallosal fibers related to the 5 functional subdivisions (regions of tongue/larynx, head/face, lower limbs, trunk and upper limbs, respectively) of the M1, were orderly identified in a proper ventro-dorsal and anterio-posterior arrangement in the topography of CC (Fig. S1). The corresponding subregions of the rostral and caudal area 7, which presented predominant inter-hemispheric connectivity in the parietal cortex, orderly placed from the isthmus to dorsal splenium of CC; and for the occipital cortices, the transcallosal fibers generally distributed in the ventral splenium in CC (Fig. 6). Besides, the commissures of temporal cortical subdivisions seemed fewer overall and in smaller pieces than the other lobes. Among them, the tracts connecting the lateral temporal neocortices mainly passed the central splenium, while the tracts from medial paleocortices traversed the ventral part.

1. **Legend of figure S1**

**Fig. S1 Regions and TDI of the transcallosal tracts** **corresponding to the primary motor cortex (M1) parcellated by the BNA.** (A) The pathways of the transcallosal tracts, corresponding to the primary motor cortex (M1) parcellated based on the BNA, were shown in 3-dimensional form in average QA space. The M1 region contained five functional subregions (regions of tongue/larynx, head/face, lower limbs, trunk and upper limbs, respectively). The transcallosal tracts were orderly identified in a proper ventro-dorsal and anterio-posterior arrangement in the topography of CC. (B and C) The subregions and TDI of fibers from M1 divisions were shown in 2-dimensional T1 sequences in MNI space and plotted at the callosal midsection in average QA space, respectively.

Abbreviations: TDI, track-density image; BNA, Human Brainnetome Atlas; M1, the primary motor cortex; CC, corpus callosum; A4tl, area 4 (tongue and larynx region); A4hf, area 4 (head and face region); A4ul, area 4 (upper limb region); A4t, area 4 (trunk region); A4ll, area 4, (lower limb region).

1. **Tables**

**Table S1** Individual information, data preprocessing and robustness tests of all 44 volunteers. The R-squared value presented the registration quality that R >60 suggested good results. The transcallosal tracts were tracked with the CC as a seed while the whole-brain fibers were tracked by global seeding, using the optimal QA threshold in each individual QA space.

| No. | Sex | Age (y) | R-squared value | Global fibers | Transcallosal tracts | Transcallosal commissural ratio (R; %) |
| --- | --- | --- | --- | --- | --- | --- |
| 1 | F | 28 | 71 | 26900 | 21926 | 81.51 |
| 2 | F | 19 | 67 | 27342 | 22513 | 82.34 |
| 3 | M | 25 | 68 | 27293 | 21051 | 77.13 |
| 4 | F | 24 | 72 | 26989 | 21434 | 79.42 |
| 5 | F | 18 | 73 | 27084 | 21496 | 79.37 |
| 6 | F | 24 | 67 | 27270 | 22134 | 81.17 |
| 7 | F | 21 | 69 | 26213 | 22732 | 86.72 |
| 8 | M | 21 | 68 | 26863 | 20626 | 76.78 |
| 9 | F | 20 | 69 | 26581 | 21194 | 79.73 |
| 10 | M | 21 | 66 | 26998 | 22494 | 83.32 |
| 11 | F | 21 | 70 | 27047 | 22626 | 83.65 |
| 12 | M | 19 | 65 | 27450 | 20576 | 74.96 |
| 13 | F | 22 | 70 | 26844 | 21892 | 81.55 |
| 14 | F | 23 | 70 | 26505 | 20073 | 75.73 |
| 15 | M | 29 | 66 | 26591 | 21848 | 82.16 |
| 16 | M | 22 | 71 | 26576 | 21848 | 82.21 |
| 17 | F | 29 | 74 | 26234 | 21805 | 83.12 |
| 18 | F | 21 | 70 | 26072 | 22545 | 86.47 |
| 19 | F | 19 | 68 | 26316 | 20942 | 79.58 |
| 20 | M | 28 | 65 | 26508 | 22744 | 85.80 |
| 21 | M | 24 | 67 | 26245 | 21167 | 80.65 |
| 22 | F | 24 | 69 | 26323 | 22743 | 86.40 |
| 23 | M | 25 | 70 | 26020 | 21804 | 83.80 |
| 24 | F | 26 | 72 | 26951 | 22498 | 83.48 |
| 25 | F | 23 | 64 | 26306 | 22669 | 86.17 |
| 26 | F | 21 | 71 | 26491 | 22609 | 85.35 |
| 27 | F | 24 | 64 | 27754 | 22289 | 80.31 |
| 28 | F | 27 | 72 | 26905 | 21282 | 79.10 |
| 29 | F | 23 | 64 | 27116 | 20455 | 75.44 |
| 30 | M | 28 | 73 | 26675 | 21756 | 81.56 |
| 31 | F | 22 | 71 | 26283 | 21546 | 81.98 |
| 32 | M | 23 | 72 | 26938 | 21316 | 79.13 |
| 33 | F | 23 | 72 | 26285 | 19561 | 74.42 |
| 34 | F | 28 | 74 | 25949 | 22768 | 87.74 |
| 35 | F | 23 | 74 | 25917 | 21683 | 83.66 |
| 36 | F | 26 | 73 | 26411 | 22497 | 85.18 |
| 37 | M | 23 | 73 | 26516 | 21894 | 82.57 |
| 38 | M | 29 | 75 | 26285 | 23371 | 88.91 |
| 39 | M | 30 | 76 | 26116 | 21969 | 84.12 |
| 40 | F | 27 | 74 | 26110 | 22798 | 87.32 |
| 41 | F | 30 | 76 | 26142 | 22663 | 86.69 |
| 42 | M | 26 | 72 | 26367 | 21312 | 80.83 |
| 43 | M | 21 | 71 | 26061 | 22234 | 85.32 |
| 44 | F | 27 | 75 | 26044 | 23385 | 89.79 |
| Averaged QA template | | | | 26007 | 21622 | 83.14 |

Abbreviations: CC, corpus callosum; M, male; F, female; QA, quantitative anisotropy.

The transcallosal commissural ratio was computed as the percentage of the transcallosal streamlines to the number of whole-brain fibers in an individual QA space (*R_i_*), while the proportion in the single averaged QA template was represented by *R_a_*.

**Table S2** The NQA values of transcallosal tracts in the AAL3 group. Considering the potential confounding effect due to the connections’ proportional differences between various lobes, we also evaluated the predominant connections in the main cortical lobes.

| Regions in AAL3 | | Cases | Mean | Standard deviation | Order of the NQA values from highest to lowest | Confidence Interval of 95% | | Min. | Max. | Amounts of tracts | Proportion (subregion: global)  (%)* | Proportion (subregion: lobe^#^)  (%)** |
| --- | --- | --- | --- | --- | --- | --- | --- | --- | --- | --- | --- | --- |
| No. | Subregions |  |  |  |  | Lower limit | Upper limit |  |  |  |  |  |
| 1 | Olfactory | 40 | 0.186 | 0.048 | 40 | 0.170 | 0.201 | 0.069 | 0.285 | 508 | 0.07 | 0.10 |
| 2 | Rectus | 43 | 0.228 | 0.045 | 38 | 0.214 | 0.242 | 0.134 | 0.338 | 7278 | 1.04 | 1.45 |
| 3 | OFCant | 40 | 0.211 | 0.051 | 41 | 0.194 | 0.227 | 0.124 | 0.344 | 734 | 0.10 | 0.15 |
| 4 | OFClat | 27 | 0.177 | 0.042 | 16 | 0.160 | 0.194 | 0.095 | 0.282 | 39 | 0.01 | 0.01 |
| 5 | OFCmed | 40 | 0.211 | 0.053 | 18 | 0.194 | 0.227 | 0.123 | 0.360 | 1044 | 0.15 | 0.21 |
| 6 | OFCpost | 43 | 0.174 | 0.031 | 24 | 0.164 | 0.183 | 0.091 | 0.235 | 329 | 0.05 | 0.07 |
| 7 | Frontal Med Orb | 44 | 0.268 | 0.048 | 25 | 0.253 | 0.283 | 0.169 | 0.355 | 30612 | 4.36 | 6.10 |
| 8 | Frontal Inf Oper | 44 | 0.298 | 0.039 | 35 | 0.286 | 0.309 | 0.196 | 0.372 | 4521 | 0.64 | 0.90 |
| 9 | Frontal Inf Orb | 37 | 0.223 | 0.050 | 29 | 0.206 | 0.240 | 0.123 | 0.325 | 199 | 0.03 | 0.04 |
| 10 | Frontal Inf Tri | 44 | 0.291 | 0.038 | 23 | 0.279 | 0.302 | 0.214 | 0.372 | 6962 | 0.99 | 1.39 |
| 11 | Frontal Mid | 44 | 0.318 | 0.041 | 7 | 0.306 | 0.331 | 0.223 | 0.398 | 126396 | 18.02 | 25.18 |
| 12 | Frontal Sup | 44 | 0.321 | 0.042 | 5 | 0.308 | 0.333 | 0.219 | 0.425 | 185907 | 26.50 | 37.03 |
| 13 | Frontal Sup Medial | 44 | 0.298 | 0.046 | 26 | 0.284 | 0.312 | 0.193 | 0.394 | 29593 | 4.22 | 5.90 |
| 14 | Supp Motor Area | 44 | 0.369 | 0.046 | 6 | 0.355 | 0.382 | 0.253 | 0.464 | 39098 | 5.57 | 7.79 |
| 15 | Precentral | 44 | 0.373 | 0.046 | 8 | 0.359 | 0.387 | 0.267 | 0.450 | 52755 | 7.52 | 10.51 |
| 16 | Rolandic Oper | 43 | 0.287 | 0.049 | 9 | 0.272 | 0.302 | 0.183 | 0.421 | 1631 | 0.23 | 0.32 |
| 17 | Paracentral Lobule | 44 | 0.369 | 0.042 | 10 | 0.357 | 0.382 | 0.266 | 0.461 | 14387 | 2.05 | 2.87 |
| 18 | Postcentral | 44 | 0.366 | 0.040 | 21 | 0.354 | 0.379 | 0.264 | 0.428 | 48379 | 6.90 | 9.64 |
| 19 | Parietal Sup | 44 | 0.353 | 0.040 | 12 | 0.341 | 0.365 | 0.249 | 0.415 | 71020 | 10.12 | 14.15 |
| 20 | Parietal Inf | 44 | 0.351 | 0.045 | 11 | 0.338 | 0.365 | 0.225 | 0.414 | 3721 | 0.53 | 0.74 |
| 21 | SupraMarginal | 44 | 0.308 | 0.040 | 1 | 0.296 | 0.320 | 0.194 | 0.391 | 2079 | 0.30 | 0.41 |
| 22 | Angular | 44 | 0.340 | 0.039 | 4 | 0.328 | 0.352 | 0.268 | 0.417 | 6185 | 0.88 | 1.23 |
| 23 | Precuneus | 44 | 0.347 | 0.050 | 14 | 0.331 | 0.362 | 0.263 | 0.482 | 9170 | 1.31 | 1.83 |
| 24 | Occipital Sup | 44 | 0.416 | 0.047 | 2 | 0.402 | 0.431 | 0.303 | 0.487 | 17587 | 2.51 | 3.50 |
| 25 | Occipital Mid | 44 | 0.373 | 0.047 | 3 | 0.359 | 0.388 | 0.269 | 0.447 | 3727 | 0.53 | 0.74 |
| 26 | Occipital Inf | 37 | 0.323 | 0.066 | 19 | 0.301 | 0.346 | 0.170 | 0.451 | 586 | 0.08 | 0.12 |
| 27 | Cuneus | 44 | 0.410 | 0.052 | 17 | 0.394 | 0.426 | 0.303 | 0.505 | 10631 | 1.52 | 2.12 |
| 28 | Calcarine | 42 | 0.373 | 0.058 | 13 | 0.355 | 0.391 | 0.213 | 0.475 | 3486 | 0.50 | 0.69 |
| 29 | Lingual | 44 | 0.313 | 0.053 | 32 | 0.297 | 0.329 | 0.203 | 0.418 | 454 | 0.06 | 0.09 |
| 30 | Heschl | 37 | 0.318 | 0.053 | 15 | 0.301 | 0.336 | 0.203 | 0.422 | 158 | 0.02 | 0.03 |
| 31 | Temporal Sup | 43 | 0.326 | 0.038 | 36 | 0.314 | 0.337 | 0.249 | 0.406 | 2598 | 0.37 | 0.52 |
| 32 | Temporal Pole Sup | 44 | 0.254 | 0.068 | 22 | 0.233 | 0.274 | 0.148 | 0.372 | 870 | 0.12 | 0.17 |
| 33 | Temporal Mid | 44 | 0.322 | 0.045 | 28 | 0.309 | 0.336 | 0.208 | 0.414 | 2183 | 0.31 | 0.43 |
| 34 | Temporal Pole Mid | 42 | 0.220 | 0.058 | 33 | 0.202 | 0.239 | 0.135 | 0.354 | 893 | 0.13 | 0.18 |
| 35 | Temporal Inf | 43 | 0.301 | 0.065 | 27 | 0.281 | 0.321 | 0.148 | 0.406 | 2582 | 0.37 | 0.51 |
| 36 | Fusiform | 43 | 0.281 | 0.061 | 31 | 0.262 | 0.300 | 0.194 | 0.443 | 1225 | 0.17 | 0.24 |
| 37 | ParaHippocampal | 44 | 0.249 | 0.061 | 20 | 0.230 | 0.268 | 0.153 | 0.384 | 330 | 0.05 | 0.07 |
| 38 | Insula | 44 | 0.282 | 0.073 | 30 | 0.260 | 0.304 | 0.156 | 0.415 | 184 | 0.03 | 0.04 |
| 39 | Cingulate Ant | 44 | 0.261 | 0.041 | 39 | 0.249 | 0.273 | 0.189 | 0.341 | 4485 | 0.64 | 0.89 |
| 40 | Cingulate Mid | 44 | 0.312 | 0.046 | 34 | 0.298 | 0.326 | 0.225 | 0.415 | 5119 | 0.73 | 1.02 |
| 41 | Cingulate Post | 44 | 0.266 | 0.058 | 37 | 0.248 | 0.284 | 0.108 | 0.461 | 1867 | 0.27 | 0.37 |

^#^ Six main lobes in the AAL3 group included the frontal lobe, temporal lobe, parietal lobe, occipital lobe, insular lobe and cingulate cortex.

* It represented the proportion of the amounts of subregional transcallosal tracts to the amounts of global tracts (including all subregions).

** It represented the proportion of the amounts of subregional transcallosal tracts to the tracts’ amounts of 6 main cortical lobes where they were (including the frontal lobe, parietal lobe, occipital lobe, temporal lobe, insular lobe and cingulate cortex).

Abbreviations: NQA, normalized quantitative anisotropy; AAL, Automated Anatomical Labeling atlas; Cingulate Ant, anterior cingulate & paracingulate gyri; Cingulate Mid, middle cingulate & paracingulate gyri; Cingulate Post; posterior cingulate gyrus; Olfactory, olfactory cortex; Rectus, gyrus rectus; OFCant, anterior orbital gyrus; OFCmed, medial orbital gyrus; OFClat, lateral orbital gyrus; OFCpost, posterior orbital gyrus; Frontal Med Orb, superior frontal gyrus, medial orbital; Frontal Inf Oper, inferior frontal gyrus, opercular part; Frontal Inf Orb, inferior frontal gyrus, pars orbitalis; Frontal Inf Tri, inferior frontal gyrus, triangular part; Frontal Mid, middle frontal gyrus; Frontal Sup, superior frontal gyrus, dorsolateral; Frontal Sup Med, superior frontal gyrus, medial; Supp Motor Area, supplementary motor area; Precentral, precentral gyrus; Rolandic Oper; Rolandic operculum; Postcentral, postcentral gyrus; Parietal Sup, superior parietal gyrus; Parietal Inf, inferior parietal gyrus, excluding supramarginal and angular gyri; SupraMarginal, supramarginal gyrus; Angular, angular gyrus; Occipital Sup, superior occipital gyrus; Occipital Mid, middle occipital gyrus; Occipital Inf, inferior occipital gyrus; Calcarine, calcarine fissure and surrounding cortex; Lingual, lingual gyrus; Heschl, Heschl’s gyrus; Temporal Sup, superior temporal gyrus; Temporal Pole Sup: temporal pole: superior temporal gyrus; Temporal Mid, middle temporal gyrus; Temporal Pole Mid, Temporal pole: middle temporal gyrus; Temporal Inf, inferior temporal gyrus; Fusiform, fusiform gyrus; ParaHippocampal, parahippocampal gyrus.

**Table S3** The subregions in the BNA and the NQA values of transcallosal tracts in the BNA group. Considering the potential confounding effect due to the connections’ proportional differences between various lobes, we also evaluated the predominant connections in the main cortical lobes.

| Regions | | Anatomical and modified Cyto-architectonic descriptions | Average NQA values* | Order of the NQA values from highest to lowest | Amounts of tracts | Proportion (subregion: global)  (%)** | Proportion (subregion: lobe^#^)  (%)*** |
| --- | --- | --- | --- | --- | --- | --- | --- |
| No. | Subregions |  |  |  |  |  |  |
| 1 | OrG_6_1 | A14m, medial area 14 | 0.240 | 81 | 12249 | 1.72 | 2.99 |
| 2 | OrG_6_2 | A12/47o, orbital area 12/47 | 0.253 | 79 | 56 | 0.01 | 0.01 |
| 3 | OrG_6_3 | A11l, lateral area 11 | 0.227 | 83 | 1433 | 0.20 | 0.35 |
| 4 | OrG_6_4 | A11m, medial area 11 | 0.235 | 82 | 57867 | 8.12 | 14.12 |
| 5 | OrG_6_5 | A13, area 13 | 0.153 | 95 | 5329 | 0.75 | 1.30 |
| 6 | OrG_6_6 | A12/47l, lateral area 12/47 | 0.276 | 75 | 166 | 0.02 | 0.04 |
| 7 | SFG_7_1 | A8m, medial area 8 | 0.383 | 18 | 6501 | 0.91 | 1.59 |
| 8 | SFG_7_2 | A8dl, dorsolateral area 8 | 0.344 | 32 | 29164 | 4.09 | 7.12 |
| 9 | SFG_7_3 | A9l, lateral area 9 | 0.321 | 49 | 39331 | 5.52 | 9.60 |
| 10 | SFG_7_4 | A6dl, dorsolateral area 6 | 0.397 | 11 | 72640 | 10.20 | 17.73 |
| 11 | SFG_7_5 | A6m, medial area 6 | 0.424 | 2 | 11483 | 1.61 | 2.80 |
| 12 | SFG_7_6 | A9m, medial area 9 | 0.345 | 31 | 1273 | 0.18 | 0.31 |
| 13 | SFG_7_7 | A10m, medial area 10 | 0.324 | 48 | 4872 | 0.68 | 1.19 |
| 14 | MFG_7_1 | A9/46d, dorsal area 9/46 | 0.311 | 60 | 23593 | 3.31 | 5.76 |
| 15 | MFG_7_2 | IFJ, inferior frontal junction | 0.281 | 72 | 2208 | 0.31 | 0.54 |
| 16 | MFG_7_3 | A46, area 46 | 0.291 | 67 | 34125 | 4.79 | 8.33 |
| 17 | MFG_7_4 | A9/46v, ventral area 9/46 | 0.266 | 76 | 10077 | 1.41 | 2.46 |
| 18 | MFG_7_5 | A8vl, ventrolateral area 8 | 0.319 | 55 | 28607 | 4.02 | 6.98 |
| 19 | MFG_7_6 | A6vl, ventrolateral area 6 | 0.313 | 59 | 15140 | 2.13 | 3.70 |
| 20 | MFG_7_7 | A10l, lateral area10 | 0.265 | 77 | 49748 | 6.98 | 12.14 |
| 21 | IFG_6_1 | A44d, dorsal area 44 | 0.245 | 80 | 270 | 0.04 | 0.07 |
| 22 | IFG_6_2 | IFS, inferior frontal sulcus | 0.283 | 70 | 804 | 0.11 | 0.20 |
| 23 | IFG_6_3 | A45c, caudal area 45 | 0.283 | 71 | 1342 | 0.19 | 0.33 |
| 24 | IFG_6_4 | A45r, rostral area 45 | 0.278 | 74 | 255 | 0.04 | 0.06 |
| 25 | IFG_6_5 | A44op, opercular area 44 | 0.302 | 63 | 67 | 0.01 | 0.02 |
| 26 | IFG_6_6 | A44v, ventral area 44 | 0.298 | 64 | 1134 | 0.16 | 0.28 |
| 27 | PrG_6_1 | A4hf, area 4(head and face region) | 0.332 | 41 | 2004 | 0.28 | 1.56 |
| 28 | PrG_6_2 | A6cdl, caudal dorsolateral area 6 | 0.381 | 20 | 21375 | 3.00 | 16.61 |
| 29 | PrG_6_3 | A4ul, area 4(upper limb region) | 0.413 | 4 | 23478 | 3.30 | 18.25 |
| 30 | PrG_6_4 | A4t, area 4(trunk region) | 0.451 | 1 | 26939 | 3.78 | 20.93 |
| 31 | PrG_6_5 | A4tl, area 4(tongue and larynx region) | 0.319 | 54 | 441 | 0.06 | 0.34 |
| 32 | PrG_6_6 | A6cvl, caudal ventrolateral area 6 | 0.287 | 68 | 1577 | 0.22 | 1.23 |
| 33 | PCL_2_1 | A1/2/3ll, area1/2/3 (lower limb region) | 0.380 | 22 | 1157 | 0.16 | 0.90 |
| 34 | PCL_2_2 | A4ll, area 4, (lower limb region) | 0.420 | 3 | 1830 | 0.26 | 1.42 |
| 35 | PoG_4_1 | A1/2/3ulhf, area 1/2/3(upper limb, head and face region) | 0.334 | 40 | 627 | 0.09 | 0.49 |
| 36 | PoG_4_2 | A1/2/3tonIa, area 1/2/3(tongue and larynx region) | 0.286 | 69 | 306 | 0.04 | 0.24 |
| 37 | PoG_4_3 | A2, area 2 | 0.386 | 14 | 2198 | 0.31 | 1.71 |
| 38 | PoG_4_4 | A1/2/3tru, area1/2/3(trunk region) | 0.410 | 5 | 46748 | 6.56 | 36.33 |
| 39 | SPL_5_1 | A7r, rostral area 7 | 0.365 | 23 | 36135 | 5.07 | 30.90 |
| 40 | SPL_5_2 | A7c, caudal area 7 | 0.328 | 45 | 16812 | 2.36 | 14.38 |
| 41 | SPL_5_3 | A5l, lateral area 5 | 0.381 | 19 | 2958 | 0.42 | 2.53 |
| 42 | SPL_5_4 | A7pc, postcentral area 7 | 0.380 | 21 | 41996 | 5.90 | 35.91 |
| 43 | SPL_5_5 | A7ip, intraparietal area 7(hIP3) | 0.337 | 38 | 3741 | 0.53 | 3.20 |
| 44 | IPL_6_1 | A39c, caudal area 39(PGp) | 0.309 | 61 | 1952 | 0.27 | 1.67 |
| 45 | IPL_6_2 | A39rd, rostrodorsal area 39(Hip3) | 0.316 | 57 | 7430 | 1.04 | 6.35 |
| 46 | IPL_6_3 | A40rd, rostrodorsal area 40(PFt) | 0.295 | 66 | 385 | 0.05 | 0.33 |
| 47 | IPL_6_4 | A40c, caudal area 40(PFm) | 0.320 | 51 | 730 | 0.10 | 0.62 |
| 48 | IPL_6_5 | A39rv, rostroventral area 39(PGa) | 0.330 | 44 | 80 | 0.01 | 0.07 |
| 49 | IPL_6_6 | A40rv, rostroventral area 40(PFop) | 0.278 | 73 | 248 | 0.03 | 0.21 |
| 50 | PCun_4_1 | A7m, medial area 7(PEp) | 0.342 | 33 | 814 | 0.11 | 0.70 |
| 51 | PCun_4_2 | A5m, medial area 5(PEm) | 0.386 | 13 | 2621 | 0.37 | 2.24 |
| 52 | PCun_4_3 | dmPOS, dorsomedial parietooccipital sulcus (PEr) | 0.331 | 42 | 445 | 0.06 | 0.38 |
| 53 | PCun_4_4 | A31, area 31 (Lc1) | 0.297 | 65 | 601 | 0.08 | 0.51 |
| 54 | MVOcC _5_1 | cLinG, caudal lingual gyrus | 0.337 | 39 | 336 | 0.05 | 0.71 |
| 55 | MVOcC _5_2 | rCunG, rostral cuneus gyrus | 0.315 | 58 | 684 | 0.10 | 1.44 |
| 56 | MVOcC _5_3 | cCunG, caudal cuneus gyrus | 0.399 | 10 | 2990 | 0.42 | 6.27 |
| 57 | MVOcC _5_4 | rLinG, rostral lingual gyrus | 0.304 | 62 | 78 | 0.01 | 0.16 |
| 58 | MVOcC _5_5 | vmPOS,ventromedial parietooccipital sulcus | 0.338 | 35 | 4348 | 0.61 | 9.12 |
| 59 | LOcC_4_1 | mOccG, middle occipital gyrus | 0.383 | 17 | 16115 | 2.26 | 33.82 |
| 60 | LOcC _4_2 | V5/MT+, area V5/MT+ | 0.316 | 56 | 4361 | 0.61 | 9.15 |
| 61 | LOcC _4_3 | OPC, occipital polar cortex | 0.403 | 7 | 1191 | 0.17 | 2.50 |
| 62 | LOcC_4_4 | iOccG, inferior occipital gyrus | 0.384 | 16 | 269 | 0.04 | 0.56 |
| 63 | LOcC _2_1 | msOccG, medial superior occipital gyrus | 0.402 | 8 | 17176 | 2.41 | 36.04 |
| 64 | LOcC _2_2 | lsOccG, lateral superior occipital gyrus | 0.389 | 12 | 107 | 0.02 | 0.22 |
| 65 | pSTS_2_1 | rpSTS, rostroposterior superior temporal sulcus | 0.406 | 6 | 14 | 0.00 | 0.22 |
| 66 | pSTS_2_2 | cpSTS, caudoposterior superior temporal sulcus | - | - | - | - | - |
| 67 | STG_6_1 | A38m, medial area 38 | 0.096 | 101 | 38 | 0.01 | 0.59 |
| 68 | STG_6_2 | A41/42, area 41/42 | 0.348 | 30 | 151 | 0.02 | 2.34 |
| 69 | STG_6_3 | TE1.0 and TE1.2 | 0.357 | 26 | 444 | 0.06 | 6.87 |
| 70 | STG_6_4 | A22c, caudal area 22 | 0.320 | 52 | 599 | 0.08 | 9.26 |
| 71 | STG_6_5 | A38l, lateral area 38 | 0.357 | 27 | 307 | 0.04 | 4.75 |
| 72 | STG_6_6 | A22r, rostral area 22 | 0.354 | 29 | 441 | 0.06 | 6.82 |
| 73 | MTG_4_1 | A21c, caudal area 21 | 0.338 | 37 | 640 | 0.09 | 9.90 |
| 74 | MTG_4_2 | A21r, rostral area 21 | 0.326 | 47 | 213 | 0.03 | 3.29 |
| 75 | MTG_4_3 | A37dl, dorsolateral area37 | 0.327 | 46 | 24 | 0.00 | 0.37 |
| 76 | MTG_4_4 | aSTS, anterior superior temporal sulcus | 0.338 | 34 | 536 | 0.08 | 8.29 |
| 77 | ITG_7_1 | A20iv, intermediate ventral area 20 | 0.196 | 87 | 52 | 0.01 | 0.80 |
| 78 | ITG_7_2 | A37elv, extreme lateroventral area37 | 0.319 | 53 | 311 | 0.04 | 4.81 |
| 79 | ITG_7_3 | A20r, rostral area 20 | 0.185 | 90 | 329 | 0.05 | 5.09 |
| 80 | ITG_7_4 | A20il, intermediate lateral area 20 | 0.331 | 43 | 185 | 0.03 | 2.86 |
| 81 | ITG_7_5 | A37vl, ventrolateral area 37 | 0.338 | 36 | 313 | 0.04 | 4.84 |
| 82 | ITG_7_6 | A20cl, caudolateral of area 20 | 0.362 | 25 | 218 | 0.03 | 3.37 |
| 83 | ITG_7_7 | A20cv, caudoventral of area 20 | 0.365 | 24 | 111 | 0.02 | 1.72 |
| 84 | FuG_3_1 | A20rv, rostroventral area 20 | 0.155 | 93 | 779 | 0.11 | 12.05 |
| 85 | FuG_3_2 | A37mv, medioventral area37 | 0.355 | 28 | 43 | 0.01 | 0.67 |
| 86 | FuG_3_3 | A37lv, lateroventral area37 | 0.320 | 50 | 438 | 0.06 | 6.77 |
| 87 | PhG_6_1 | A35/36r, rostral area 35/36 | 0.136 | 97 | 11 | 0.00 | 0.17 |
| 88 | PhG_6_2 | A35/36c, caudal area 35/36 | 0.222 | 85 | 8 | 0.00 | 0.12 |
| 89 | PhG_6_3 | TL, area TL (lateral PPHC, posterior parahippocampal gyrus) | 0.188 | 89 | 29 | 0.00 | 0.45 |
| 90 | PhG_6_4 | A28/34, area 28/34 (EC, entorhinal cortex) | 0.097 | 100 | 10 | 0.00 | 0.15 |
| 91 | PhG_6_5 | TI, area TI (temporal agranular insular cortex) | 0.118 | 98 | 176 | 0.02 | 2.72 |
| 92 | PhG_6_6 | TH, area TH (medial PPHC) | 0.194 | 88 | 46 | 0.01 | 0.71 |
| 93 | INS_6_1 | G, hypergranular insula | 0.401 | 9 | 18 | 0.00 | 27.69 |
| 94 | INS_6_2 | vIa, ventral agranular insula | 0.116 | 99 | 8 | 0.00 | 12.31 |
| 95 | INS_6_3 | dIa, dorsal agranular insula | - | - | - | - | - |
| 96 | INS_6_4 | vId/vIg, ventral dysgranular and granular insula | 0.385 | 15 | 39 | 0.01 | 60.00 |
| 97 | INS_6_5 | dIg, dorsal granular insula | - | - | - | - | - |
| 98 | INS_6_6 | dId, dorsal dysgranular insula | - | - | - | - | - |
| 99 | CG_7_1 | A23d, dorsal area 23 | 0.226 | 84 | 212 | 0.03 | 7.81 |
| 100 | CG_7_2 | A24rv, rostroventral area 24 | 0.219 | 86 | 504 | 0.07 | 18.58 |
| 101 | CG_7_3 | A32p, pregenual area 32 | 0.170 | 91 | 72 | 0.01 | 2.65 |
| 102 | CG_7_4 | A23v, ventral area 23 | 0.255 | 78 | 291 | 0.04 | 10.73 |
| 103 | CG_7_5 | A24cd, caudodorsal area 24 | 0.154 | 94 | 219 | 0.03 | 8.07 |
| 104 | CG_7_6 | A23c, caudal area 23 | 0.165 | 92 | 168 | 0.02 | 6.19 |
| 105 | CG_7_7 | A32sg, subgenual area 32 | 0.137 | 96 | 1247 | 0.18 | 45.96 |

Abbreviations: NQA, normalized quantitative anisotropy; BNA, Human Brainnetome Atlas; OrG, orbital gyrus; SFG, superior frontal gyrus; MFG, middle frontal gyrus; IFG, inferior frontal gyrus; PrG, precentral gyrus; PCL, paracentral lobule; PoG, postcentral gyrus; SPL, superior parietal lobe; IPL, inferior parietal lobe; PCun, precuneus; LOcC, lateral occipital cortex; MVOcC, medioventral occipital cortex; pSTS, posterior superior temporal sulcus; STG, superior temporal gyrus; MTG, middle temporal gyrus; ITG, inferior temporal gyrus; FuG, fusiform gyrus; PhG, parahippocampal gyrus; INS, insula; CG, cingulate gyrus;

^#^ Seven main lobes in the BNA group including the prefrontal lobe, central region, temporal lobe, parietal lobe, occipital lobe, insular lobe and cingulate cortex.

* The average NQA values represented the NQA values of the merged transcallosal tracts across all 44 cases measured in the average QA space.

** It represented the proportion of the amounts of subregional transcallosal tracts to the amounts of global tracts (including all subregions).

*** It represented the proportion of the amounts of subregional transcallosal tracts to the tracts’ amounts of 7 main cortical lobes where they were (including the prefrontal lobe, central region, parietal lobe, occipital lobe, temporal lobe, insula and cingulate cortex).

**Table S4** Results of the paired sample tests and ICC tests. The paired sample tests (including paired t-test and Wilcoxon test) were between the NQA values of transcallosal tracts in the AAL3 and BNA groups.

| 19 main coupled regions | Components of the transcallosal tracts in the 19 main regions | | NQA values* | | Paired sample tests  (t-test^a^ or Wilcoxon test^b^) | *P* value | ICC |
| --- | --- | --- | --- | --- | --- | --- | --- |
|  | AAL3 | BNA | AAL3 | BNA |  |  |  |
| OFC | Olfactory, Rectus, OFCant, OFClat, OFCmed, OFCpost, Frontal Med Orb, Frontal Inf Orb | OrG (1-6) | 0.262±0.047 | 0.264±0.045 | -1.326^a^ | 0.192 | 0.985^**^ |
| SFG | Frontal Sup, Frontal Sup Medial, Supp Motor Area | SFG (1-7), MFG 7 | 0.328±0.041 | 0.329±0.042 | -0.257^b^ | 0.797 | 0.985^**^ |
| MFG | Frontal Mid | MFG (1-6) | 0.318±0.041 | 0.318±0.041 | -0.012^b^ | 0.991 | 0.983^**^ |
| IFG | Frontal Inf Oper, Frontal Inf Tri | IFG (1-6) | 0.295±0.036 | 0.289±0.039 | -1.517^b^ | 0.129 | 0.853^**^ |
| PrG | Precentral, Rolandic Oper | PrG (1-6) | 0.379±0.044 | 0.383±0.044 | -0.934^b^ | 0.351 | 0.940^**^ |
| PCL | Paracentral Lobule | PCL (1-2) | 0.369±0.042 | 0.364±0.045 | -1.786^b^ | 0.074 | 0.856^**^ |
| PoG | Postcentral | PoG (1-4) | 0.366±0.040 | 0.368±0.042 | -1.821^a^ | 0.076 | 0.989^**^ |
| SPL | Parietal Sup | SPL (1-5) | 0.353±0.040 | 0.354±0.041 | -1.132^b^ | 0.258 | 0.983^**^ |
| IPL | Parietal Inf, SupraMarginal, Angular | IPL (1-6) | 0.339±0.040 | 0.335±0.034 | -1.645^b^ | 0.100 | 0.874^**^ |
| Pcun | Precuneus | PCun (1-4) | 0.349±0.054 | 0.344±0.047 | 0.830^a^ | 0.411 | 0.582^**^ |
| LOcC | Occipital Sup, Occipital Mid, Occipital Inf | LOcC (1-6) | 0.407±0.047 | 0.405±0.048 | 0.974^a^ | 0.336 | 0.962^**^ |
| MVOcC | Cuneus, Calcarine, Lingual | MVOcC (1-5) | 0.393±0.059 | 0.387±0.053 | 1.060^a^ | 0.295 | 0.748^**^ |
| STG | Heschl, Temporal Sup, Temporal Pole Sup | pSTS 1, STG (1-6) | 0.307±0.046 | 0.310±0.047 | -0.782^b^ | 0.434 | 0.774^**^ |
| MTG | Temporal Mid, Temporal Pole Mid | MTG (1-4) | 0.289±0.057 | 0.295±0.060 | -1.377^b^ | 0.168 | 0.863^**^ |
| ITG | Temporal Inf | ITG (1-7) | 0.299±0.064 | 0.284±0.064 | -1.937^b^ | 0.053 | 0.862^**^ |
| FuG | Fusiform | FuG (1-3) | 0.277±0.064 | 0.267±0.067 | -1.622^b^ | 0.105 | 0.878^**^ |
| PhG | ParaHippocampal | PhG (1-6) | 0.248±0.061 | 0.230±0.051 | -1.540^b^ | 0.123 | 0.572^**^ |
| INS | Insula | INS (1, 2,4) | 0.282±0.073 | 0.279±0.071 | -1.109^b^ | 0.268 | 0.779^**^ |
| CG | Cingulate Ant, Cingulate Mid, Cingulate Post | CG (1-7) | 0.302±0.039 | 0.314±0.049 | -1.770^a^ | 0.084 | 0.488^**^ |

Abbreviations: NQA, normalized quantitative anisotropy; AAL, Automated Anatomical Labeling atlas; BNA, Human Brainnetome Atlas; ICC, intraclass correlation coefficient; OFC, orbitofrontal cortex; SFG, superior frontal gyrus; MFG, middle frontal gyrus; IFG, inferior frontal gyrus; PrG, precentral gyrus; PCL, paracentral lobule; PoG, postcentral gyrus; SPL, superior parietal lobe; IPL, inferior parietal lobe; Pcun, precuneus; LOcC, lateral occipital cortex; MVOcC, medioventral occipital cortex; STG, superior temporal gyrus; MTG, middle temporal gyrus; ITG, inferior temporal gyrus; FuG, fusiform gyrus; PhG, parahippocampal gyrus; INS, insula; CG, cingulate gyrus; Cingulate Ant, anterior cingulate & paracingulate gyri.

a Since the NQA values of the transcallosal tracts in both AAL3 and BNA groups were in normal distribution without outliers, we performed a paired t-test and “a” represented the t-value.

b Since the NQA values of the transcallosal tracts were in abnormal distribution, we performed a Wilcoxon test and “b” represented the Z-score.

* NQA values represented that the averaged NQA values of the transcallosal tracts which were respectively parcellated based on the AAL3 and BNA across all 44 cases in individual QA spaces.

** It indicated that P＜0.0001 in ICC test with a two-way random model.

**Table S5** The segmentation and components of seven cortical subregions and the NQA values of the corresponding transcallosal tracts across all 44 cases.

| Cortical subregions | | | Mean | Standard deviation | Confidence Interval of 95% | | Min. | Max. |
| --- | --- | --- | --- | --- | --- | --- | --- | --- |
| No. | - | Components |  |  | Lower limit | Upper limit |  |  |
| 1 | OFC | Olfactory, Rectus, OFCant, OFClat, OFCmed, OFCpost, Frontal Med Orb | 0.258 | 0.047 | 0.244 | 0.273 | 0.167 | 0.343 |
| 2 | Prefrontal cortex | Frontal Sup, Frontal Sup Medial, Supp Motor Area, Frontal Mid, Frontal Inf Oper, Frontal Inf Tri, Frontal Inf Orb, Cingulate Ant | 0.316 | 0.040 | 0.304 | 0.328 | 0.220 | 0.414 |
| 3 | Central region | Precentral, Rolandic Oper, Paracentral Lobule, Postcentral, Cingulate Mid | 0.368 | 0.041 | 0.356 | 0.381 | 0.271 | 0.443 |
| 4 | Parietal lobe | Parietal Sup, Parietal Inf, SupraMarginal, Angular, Precuneus, Cingulate Post | 0.349 | 0.039 | 0.337 | 0.361 | 0.253 | 0.407 |
| 5 | Occipital lobe | Occipital Sup, Occipital Mid, Occipital Inf, Cuneus, Calcarine, Lingual | 0.406 | 0.047 | 0.392 | 0.421 | 0.293 | 0.484 |
| 6 | Temporal lobe | Heschl, Temporal Sup, Temporal Pole Sup, Temporal Mid, Temporal Pole Mid, Temporal Inf, Fusiform, ParaHippocampal | 0.291 | 0.051 | 0.275 | 0.306 | 0.197 | 0.388 |
| 7 | Insula | Insula | 0.282 | 0.073 | 0.260 | 0.304 | 0.156 | 0.415 |

Abbreviations: NQA, normalized quantitative anisotropy; OFC, orbitofrontal cortex; Cingulate Ant, anterior cingulate & paracingulate gyri; Cingulate Mid, middle cingulate & paracingulate gyri; Cingulate Post; posterior cingulate gyrus; Olfactory, olfactory cortex; Rectus, gyrus rectus; OFCant, anterior orbital gyrus; OFCmed, medial orbital gyrus; OFClat, lateral orbital gyrus; OFCpost, posterior orbital gyrus; Frontal Med Orb, superior frontal gyrus, medial orbital; Frontal Inf Oper, inferior frontal gyrus, opercular part; Frontal Inf Orb, inferior frontal gyrus, pars orbitalis; Frontal Inf Tri, inferior frontal gyrus, triangular part; Frontal Mid, middle frontal gyrus; Frontal Sup, superior frontal gyrus, dorsolateral; Frontal Sup Med, superior frontal gyrus, medial; Supp Motor Area, supplementary motor area; Precentral, precentral gyrus; Rolandic Oper; Rolandic operculum; Postcentral, postcentral gyrus; Parietal Sup, superior parietal gyrus; Parietal Inf, inferior parietal gyrus, excluding supramarginal and angular gyri; SupraMarginal, supramarginal gyrus; Angular, angular gyrus; Occipital Sup, superior occipital gyrus; Occipital Mid, middle occipital gyrus; Occipital Inf, inferior occipital gyrus; Calcarine, calcarine fissure and surrounding cortex; Lingual, lingual gyrus; Heschl, Heschl’s gyrus; Temporal Sup, superior temporal gyrus; Temporal Pole Sup: temporal pole: superior temporal gyrus; Temporal Mid, middle temporal gyrus; Temporal Pole Mid, Temporal pole: middle temporal gyrus; Temporal Inf, inferior temporal gyrus; Fusiform, fusiform gyrus; ParaHippocampal, parahippocampal gyrus.

**Table S6** The results of multiple comparisons test for the NQA values of the seven main bundles across all 44 cases.

| Sidak's multiple comparisons test | Mean difference | 95.00% CI of difference | Significance | Summary | Adjusted *P* value |
| --- | --- | --- | --- | --- | --- |
| orbitofrontal vs. prefrontal | -0.05794 | -0.09009 to -0.02579 | Yes | **** | <0.0001 |
| orbitofrontal vs. central | -0.1099 | -0.1420 to -0.07774 | Yes | **** | <0.0001 |
| orbitofrontal vs. parietal | -0.09052 | -0.1227 to -0.05837 | Yes | **** | <0.0001 |
| orbitofrontal vs. occipital | -0.1481 | -0.1803 to -0.1160 | Yes | **** | <0.0001 |
| orbitofrontal vs. temporal | -0.03235 | -0.06450 to -0.0001942 | Yes | * | 0.0472 |
| orbitofrontal vs. insular | -0.0235 | -0.05565 to 0.008654 | No | ns | 0.4276 |
| prefrontal vs. central | -0.05195 | -0.08411 to -0.01980 | Yes | **** | <0.0001 |
| prefrontal vs. parietal | -0.03258 | -0.06474 to -0.0004283 | Yes | * | 0.0439 |
| prefrontal vs. occipital | -0.09019 | -0.1223 to -0.05803 | Yes | **** | <0.0001 |
| prefrontal vs. temporal | 0.02559 | -0.006563 to 0.05774 | No | ns | 0.2807 |
| prefrontal vs. insular | 0.03444 | 0.002285 to 0.06659 | Yes | * | 0.0246 |
| central vs. parietal | 0.01937 | -0.01278 to 0.05152 | No | ns | 0.7645 |
| central vs. occipital | -0.03823 | -0.07039 to -0.006081 | Yes | ** | 0.0069 |
| central vs. temporal | 0.07754 | 0.04539 to 0.1097 | Yes | **** | <0.0001 |
| central vs. insular | 0.08639 | 0.05424 to 0.1185 | Yes | **** | <0.0001 |
| parietal vs. occipital | -0.0576 | -0.08976 to -0.02545 | Yes | **** | <0.0001 |
| parietal vs. temporal | 0.05817 | 0.02602 to 0.09033 | Yes | **** | <0.0001 |
| parietal vs. insular | 0.06702 | 0.03487 to 0.09917 | Yes | **** | <0.0001 |
| occipital vs. temporal | 0.1158 | 0.08362 to 0.1479 | Yes | **** | <0.0001 |
| occipital vs. insular | 0.1246 | 0.09247 to 0.1568 | Yes | **** | <0.0001 |
| temporal vs. insular | 0.008848 | -0.02331 to 0.04100 | No | ns | >0.9999 |

Abbreviations: CI, confidence interval.
