# Supplementary figures and images for: Mapping Human Corpus Callosum Connectivity With Diffusion Spectrum Imaging: A Deterministic Tractography Approach
Source: Brain Behav. 2026 Mar 12;16(3):e71306. doi: 10.1002/brb3.71306 (PMC13093777; doi:10.1002/brb3.71306)

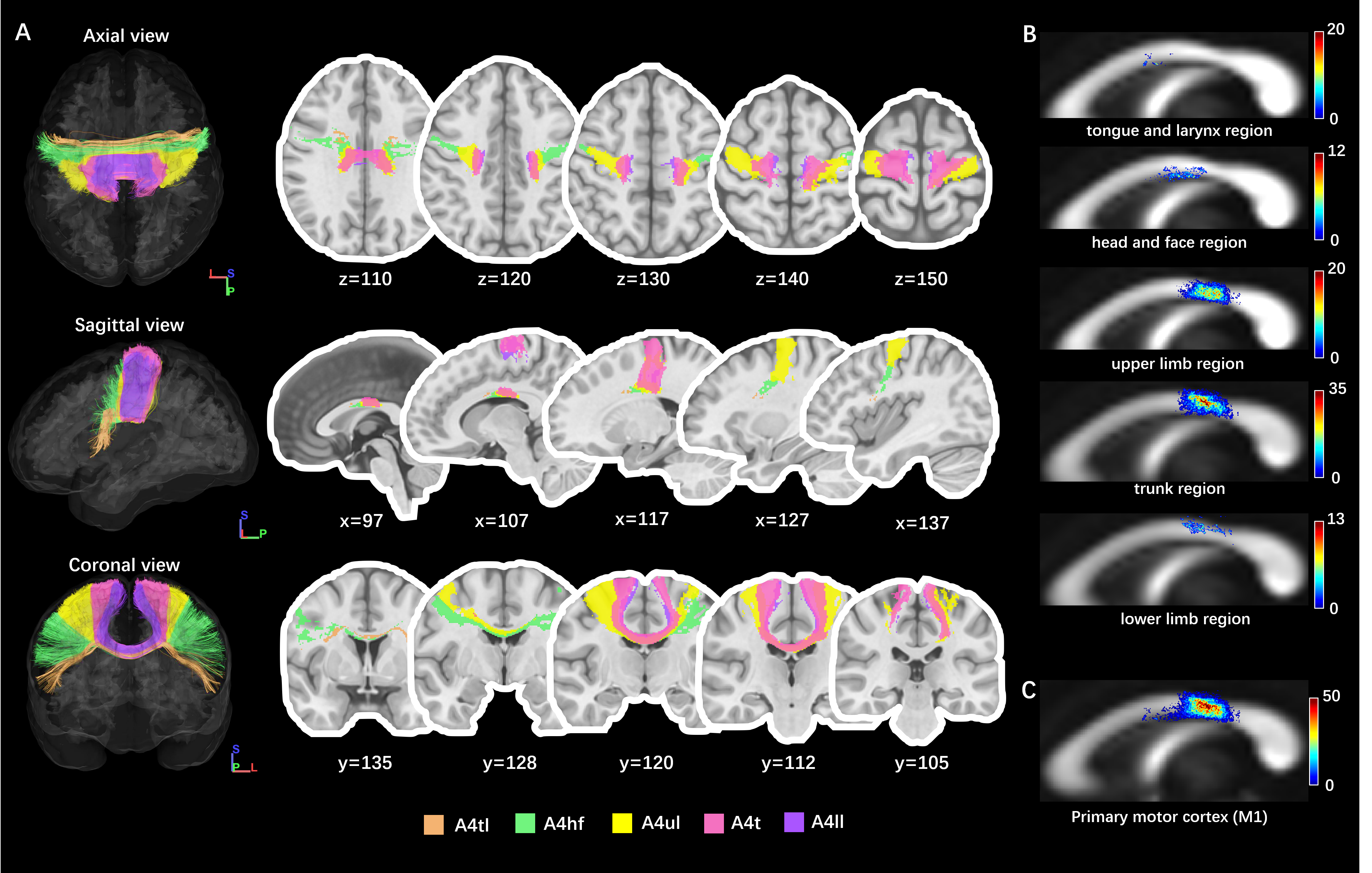

Supplement: Supplementary file 2 — Supplementary FigureS1: brb371306‐sup‐0002‐FigureS1.tif [file BRB3-16-e71306-s001.tif]
